# Children at onset of type 1 diabetes show altered *N*-glycosylation of plasma proteins and IgG

**DOI:** 10.1007/s00125-022-05703-8

**Published:** 2022-05-27

**Authors:** Najda Rudman, Domagoj Kifer, Simranjeet Kaur, Vesna Simunović, Ana Cvetko, Flemming Pociot, Grant Morahan, Olga Gornik

**Affiliations:** 1 Faculty of Pharmacy and Biochemistry, University of Zagreb, Zagreb, Croatia; 2 Steno Diabetes Center Copenhagen, Herlev, Denmark; 3 Faculty of Health and Medical Sciences, University of Copenhagen, Copenhagen, Denmark; 4 Copenhagen Diabetes Research Center (CPH-DIRECT), Department of Pediatrics E, Herlev Hospital, Herlev, Denmark; 5 Centre for Diabetes Research, The Harry Perkins Institute for Medical Research, Perth, WA Australia; 6 University of Melbourne, Parkville, VIC Australia

**Keywords:** IgG, *N-*glycans, Plasma proteins, Predictive model, Type 1 diabetes onset

## Abstract

**Aims/hypothesis:**

Individual variation in plasma *N*-glycosylation has mainly been studied in the context of diabetes complications, and its role in type 1 diabetes onset is largely unknown. Our aims were to undertake a detailed characterisation of the plasma and IgG *N*-glycomes in patients with recent onset type 1 diabetes, and to evaluate their discriminative potential in risk assessment.

**Methods:**

In the first part of the study, plasma and IgG *N*-glycans were chromatographically analysed in a study population from the DanDiabKids registry, comprising 1917 children and adolescents (0.6–19.1 years) who were newly diagnosed with type 1 diabetes. A follow-up study compared the results for 188 of these participants with those for their 244 unaffected siblings. Correlation of *N*-glycan abundance with the levels and number of various autoantibodies (against IA-2, GAD, ZnT8R, ZnT8W), as well as with sex and age at diagnosis, were estimated by using general linear modelling. A disease predictive model was built using logistic mixed-model elastic net regression, and evaluated using a 10-fold cross-validation.

**Results:**

Our study showed that onset of type 1 diabetes was associated with an increase in the proportion of plasma and IgG high-mannose and bisecting GlcNAc structures, a decrease in monogalactosylation, and an increase in IgG disialylation. ZnT8R autoantibody levels were associated with higher IgG digalactosylated glycan with bisecting GlcNAc. Finally, an increase in the number of autoantibodies (which is a better predictor of progression to overt diabetes than the level of any individual antibody) was accompanied by a decrease in the proportions of some of the highly branched plasma *N*-glycans. Models including age, sex and *N-*glycans yielded notable discriminative power between children with type 1 diabetes and their healthy siblings, with AUCs of 0.915 and 0.869 for addition of plasma and IgG *N-*glycans, respectively.

**Conclusions/interpretation:**

We defined *N-*glycan changes accompanying onset of type 1 diabetes, and developed a predictive model based on *N-*glycan profiles that could have valuable potential in risk assessment. Increasing the power of tests to identify individuals at risk of disease development would be a considerable asset for type 1 diabetes prevention trials.

**Graphical abstract:**

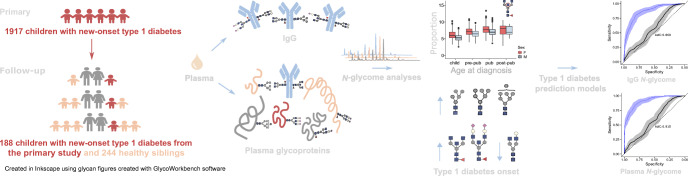

**Supplementary Information:**

The online version of this article (10.1007/s00125-022-05703-8) contains peer-reviewed but unedited supplementary material.

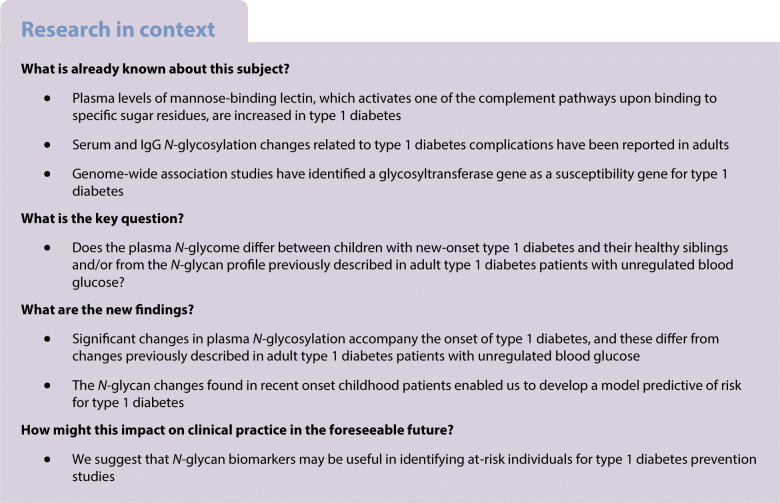



## Introduction

Type 1 diabetes is a chronic autoimmune disease with an unknown cause, and is marked by destruction of insulin-producing pancreatic beta cells [1]. The number of children and adolescents diagnosed with type 1 diabetes is increasing at an annual rate of approximately 3% [2]. Although measurement of islet autoantibodies can expose the disease years before clinical diagnosis, continuous monitoring is expensive, is difficult in young children and is not adequately sensitive or specific in adults [3, 4]. As early identification of type 1 diabetes can minimise morbidity and facilitate prevention, development of risk assessment tools is an important task, and many trials, initiatives and networks have been established with this aim [5]*.* A recently established genetic risk score showed good discriminative values [6], but identification of additional biomarkers that could contribute to the risk assessment would be of great value.

*N-*glycosylation of plasma proteins is a strictly regulated and very complex enzymatic process by which different oligosaccharides are added to protein backbones [7], modulating protein function in many instances [8]. Glycosylation must not be confused with glycation, which is a non-enzymatic reaction, such as described for HbA_1c_ [9]. Detailed genetic studies have identified genes that are important in regulating the type of glycans added, and also showed that residues flanking the *N-*glycosylation motif affect the added glycan type [10]. The human plasma *N-*glycome is remarkably stable within an individual under physiological conditions [11], and yet is very sensitive to various pathological processes, thus enabling consideration of *N-*glycans as diagnostic and prognostic markers [12–14]. Diabetes classification may be difficult as it is dependent on conditions at the time of diagnosis; for example, some individuals diagnosed with type 2 diabetes have islet autoantibodies [15]. We showed previously that it is possible to differentiate between diabetes types and even identify individuals at an increased risk of developing type 2 diabetes in the future based on their *N-*glycan profiles [12, 13, 16, 17]. *N-*glycosylation profiling may also prove to be an asset in comparison with antibody testing due to cost reduction, which may be further reduced upon determination of diagnostically significant *N-*glycan structures.

During the process of eukaryotic protein *N-*glycosylation, a block of 14 sugars is transferred cotranslationally to specific asparagine residues in the endoplasmic reticulum [18], and afterwards modified in the Golgi complex. This results in numerous modifications, such as branching, fucosylation, sialylation, etc. [19]. Under physiological conditions, approximately 3% of glucose is used in the hexosamine biosynthesis pathway [20], in which the donor molecule for the process of *N-*glycosylation, uridine diphosphate-*N-*acetylglucosamine (UDP-GlcNAc), is synthesised [18]. The degree of glycan branching that defines glycan complexity (biantennary, triantennary and tetraantennary glycans) depends on the UDP-GlcNAc availability [19]. Elevated glucose flux through the hexosamine biosynthesis pathway results in elevated levels of UDP-GlcNAc and potentially highly branched glycans [19, 21]. We previously reported that complex highly branched serum *N-*glycans were increased in adult type 1 diabetes patients with unregulated blood glucose [22].

In addition, plasma levels of mannose-binding lectin, which activates one of the complement pathways upon binding to specific sugar residues [23], were increased in a type 1 diabetes population [24]. Aberrant *N-*glycosylation of T cell proteins was implicated in the type 1 diabetes onset [14]. Genome-wide association studies identified the glycosyltransferase gene encoding fucosyltransferase 2 as one of the susceptibility genes for type 1 diabetes [25].

Therefore, we undertook the current study to identify plasma *N-*glycans characteristic of the early phase of type 1 diabetes by comparing children with type 1 diabetes and their healthy siblings, to compare these results with the *N-*glycan profile previously described in adult type 1 diabetes patients with unregulated blood glucose, and to describe age- and sex-dependent changes in the plasma *N-*glycome in children and adolescents with type 1 diabetes. As far as we are aware, this is the first study of plasma *N-*glycosylation changes at the onset of type 1 diabetes. We hypothesise that plasma *N-*glycosylation may differ between children newly diagnosed with type 1 diabetes and their healthy siblings, and thus could contribute to type 1 diabetes risk assessment.

## Methods

### Ethics statement

The study was approved by the ethics committee of the University of Zagreb, Faculty of Pharmacy and Biochemistry, and the Danish Regional Ethical Committee (KA-95139 m). The study was performed in accordance with the Declaration of Helsinki. Informed consent was obtained from all the patients and if the participant was under 18 years consent from the parents/guardians was obtained.

### Study participants

In the primary study, plasma samples from 1917 children and adolescents with type 1 diabetes (median age 10.2 years, range 0.6–19.1 years), collected within 3 months of disease diagnosis through the Danish Registry of Childhood and Adolescent Diabetes (DanDiabKids) [26], were analysed. The study population was divided based on sex and into four age categories (very young children, pre-pubertal children, pubertal children and post-pubertal children). Pubertal status was defined based on age, and different cut-offs were used for pubertal onset/end of puberty for boys and girls. Information on levels of autoantibodies were available for 300 study participants, and included levels of islet autoantibodies against arginine zinc transporter 8 (ZnT8R), tryptophan zinc transporter 8 (ZnT8W), GAD and insulinoma-associated protein 2 (IA-2).

The follow-up family-based study comprised plasma samples from 188 of the 1917 participants involved in the primary study which were reanalysed, and 244 unaffected siblings (median age 11 years, range 2–23 years), collected through the same registry. The availability of the sample for the affected sibling was the means by which the unaffected siblings were identified for inclusion in the registry, rather than the converse. For some affected individuals, multiple siblings were included in the study (from one to five per affected individual), but in the majority of cases, a sample was only available for one unaffected sibling. More than 95% of the sibling samples were collected at the same date as the proband sample, and the sampling dates were quite equally distributed over the year. The year of sampling for unaffected siblings ranged from 1997 to 2000, and the last registry data extraction and disease status check for unaffected siblings was performed in January 2019. Some of the unaffected siblings were lost for follow-up if they were diagnosed above the age of 18 years, because at this age they are often referred to adult type 1 diabetes clinics. At the last disease status check, it was established that two formerly unaffected individuals developed type 1 diabetes, one within 6 years and the other within 9 years.

### *N-*Glycan analyses

**Fig. 1:**
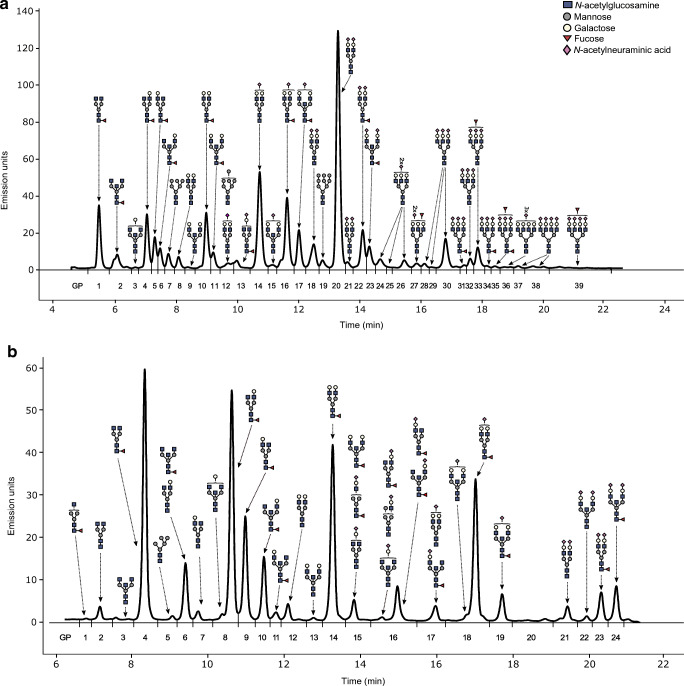
Example of a chromatogram of *N*-glycans released from total plasma proteins (**a**) and IgG (**b**). Blue squares, *N*-acetylglucosamine (GlcNAc); grey circles, mannose; yellow circles, galactose; red triangles, fucose; purple diamonds, *N*-acetylneuraminic acid (sialic acid). GP, glycan peak. The figure was created in Inkscape (https://inkscape.org/) using glycan figures created using GlycoWorkbench software [52]

Before the analyses, samples were randomised throughout the multiwell plates. To minimise experimental error, standard samples were added to each plate. A 10 μl aliquot of plasma was used for *N-*glycan profiling of total plasma proteins, whereas 70 μl of plasma was used as the starting material to perform IgG isolation using a protein G monolithic plate (BIA Separations, Slovenia) [27]. *N-*glycans on both IgG and plasma proteins were afterwards released and labelled as described previously [28]. Hydrophilic interaction ultra-performance liquid chromatography (HILIC-UPLC) on a Waters Acquity UPLC instrument (Milford, MA, USA) was used to separate fluorescently labelled *N-*glycans, as reported previously [27]. Automated integration [29] was applied to separate the chromatograms into 24 peaks for IgG *N-*glycans (GP1–GP24) and 39 peaks for plasma *N-*glycans (GP1–GP39) (Fig. 1, ESM Tables 1 and 2). ESM Tables 1 and 2 list the detected peaks for the most abundant glycan structures. The amount of glycans in each peak was expressed as a percentage of total integrated area. In addition to directly measured chromatographic peaks, nine IgG and 15 plasma derived traits representing specific glycosylation features were calculated (ESM Tables 3 and 4).

### Measurements of islet autoantibodies

Islet autoantibodies against ZnT8R, ZnT8W, GAD and IA-2 were measured as described previously [30, 31].

### Statistical analysis

Each peak in the chromatograms obtained from UPLC analysis was normalised by total chromatogram area, log-transformed and then batch-corrected using ComBat method (R package *sva* [32]). Before any statistical modelling, percentages of *N-*glycan peaks were standardised to a standard normal distribution using inverse transformation of ranks to normality (R package *GenABEL* [33]). Estimation of sex and age effect on the *N-*glycome of children with type 1 diabetes was performed using general linear modelling with glycan area as the dependent variable, and sex (levels: male or female) crossed with age group (ordered levels: child, pre-puberty, puberty or post-puberty) as independent variables. Post hoc pairwise comparisons within same sex or same age group were performed using two tailed *t* test (R package *emmeans* [34]). The false discovery rate for all tests was controlled using the Benjamini–Hochberg method [35]. For children with type 1 diabetes and known level of autoantibodies, the change in glycosylation was estimated using a linear model with glycan area as the dependent variable and antibody level (*c*_ab_) as the independent variable, accounting for levels higher than the limit of quantification (LOQ_ab_) by using an indicator variable (*I*):

$$ \mathrm{glycan}\left({c}_{\mathrm{ab}},I\right)={b}_0+I\times {b}_1\times {c}_{\mathrm{ab}}+\left(1-I\right)\times {b}_2\kern0.5em I=\left\{\begin{array}{c}1\ \mathrm{if}\ {c}_{\mathrm{ab}}< LO{Q}_{\mathrm{ab}}\\ {}0\ \mathrm{if}\ {c}_{\mathrm{ab}}\ge {LOQ}_{\mathrm{ab}}\end{array}\right. $$

where *b*_0_ is the intercept, and *b*_1_ and *b*_2_ are estimated effects of autoantibody level on glycan abundance. The analysis was adjusted for sex and age. Change in glycosylation was also estimated using a linear model with glycan area as the dependent variable, and the number of autoantibodies (ordered levels: 1, 2, 3 or 4), sex and age as independent variables. The inter-relationship of type 1 diabetes status and *N-*glycome was estimated for all siblings using logistic mixed-model elastic net regression (*α* = 0.1, *λ* = 10^−4^), by comparing the AUC of two receiver operating characteristic curves obtained from two models (R packages *glmnet* [36] and *pROC* [37]): (1) a full model (with disease status as the dependent variable; sex, age and all standardised glycan peaks as independent fixed variables; and family ID as random variable), and (2) a null model, which was the same as the full model but without glycan peaks. To avoid overfitting, a 10-fold cross-validation was used. Estimated AUCs of receiver operating characteristic curves were compared using bootstrapping (2000 replicates).

All statistical analysis was performed using R programming software (version 3.5.2) [38].

## Results

### Study population and *N-*glycan proportions

A description of the research population is provided in Table 1. The distributions of plasma and IgG *N-*glycans of children with type 1 diabetes from the primary study are shown in ESM Table 5. The *N-*glycan profiles of children with type 1 diabetes and their unaffected siblings from the follow-up family-based study, as well as the two formerly unaffected siblings who eventually developed the disease, are presented in ESM Tables 6 and 7, and ESM Figs 1 and 2. Intercorrelation of the assessed *N-*glycans across participants with type 1 diabetes is shown in ESM Figs 3 and 4. Significant differences between studied groups were observed for several *N-*glycome features (Tables 2 and 3).

**Table 1:** Description of the research population

	Primary study (children with type 1 diabetes)				Primary with measured islet autoantibodies^a^				Follow-up family-based study	
Variable	Very young	Pre-pubertal	Pubertal	Post-pubertal	1	2	3	4	Healthy siblings	Type 1 diabetes
Number of participants	391	918	550	58	13	34	72	181	244	188
Number of female participants	192	352	312	48	8	14	28	86	116	86
Age of female participants (years)	4.2 (0.6–6)	8.8 (6–11)	12.6 (11–15)	16 (15–19.1)	12.7 (6.9–14.5)	10.7 (2.3–14.4)	9.2 (4.7–14.9)	9.7 (0.6–16.1)	11 (2–23)	9.7 (0.6–16.1)
Number of male participants	199	566	238	10	5	20	44	95	128	102
Age of male participants (years)	4 (0.6–6)	10.3 (6–13)	14.3 (13–16.9)	17.4 (17–18.3)	10.2 (5.4–11.8)	11.4 (2–15.9)	11.5 (2.4–17.6)	10.7 (2.6–16.1)	11 (2–23)	10.1 (1.4–16.9)

Values for age are medians (range)

^a^Grouped by number of autoantibodies

**Table 2:** Associations of the directly measured and derived plasma *N*-glycans with disease status, further adjusted for age and sex, and corrected for multiple testing

Glycan	Description^a^	OR	95% CI	SE	*p* value
GP2	FA2B glycan	2.09	1.58, 2.76	0.30	1.16 × 10^−5^
GP4	FA2[6]G1 glycan	0.60	0.46, 0.79	0.08	2.16 × 10^−3^
GP5	FA2[3]G1 glycan	0.64	0.47, 0.86	0.10	1.32 × 10^−2^
GP7	M6 glycan	1.69	1.28, 2.24	0.24	2.01 × 10^−3^
GP10	FA2G2 glycan	0.57	0.43, 0.76	0.08	1.24 × 10^−3^
GP12	M7 glycan and A2G2S1 glycan	1.86	1.37, 2.53	0.29	1.15 × 10^−3^
GP17	FA2BG2S1 glycan	1.47	1.08, 2.00	0.23	5.04 × 10^−2^
GP21	A2G2S2 glycan	1.52	1.16, 1.98	0.21	8.56 × 10^−3^
GP22	FA2G2S2 glycan	1.74	1.32, 2.29	0.25	1.15 × 10^−3^
GP23	FA2BG2S2 glycan	1.66	1.23, 2.25	0.26	5.61 × 10^−3^
GP25	A3G3S2 glycan	2.12	1.56, 2.87	0.33	4.54 × 10^−5^
GP29	A3G3S3 glycan	1.62	1.20, 2.19	0.25	7.12 × 10^−3^
B	Structures with bisecting GlcNAc	1.72	1.26, 2.34	0.27	3.52 × 10^−3^
G1	Monogalactosylated structures	0.63	0.47, 0.84	0.09	7.01 × 10^−3^
HM	High-mannose structures	1.65	1.26, 2.16	0.23	2.16 × 10^−3^

Only statistically significant associations are presented; for all associations see electronic supplementary material [ESM], Table 9

^a^The description relates to the percentage of that glycan in total plasma *N*-glycans

GP, glycan peak. Structure abbreviations: all *N*-glycans have two core GlcNAcs; F at the start of the abbreviation indicates a core fucose α1,6-linked to the inner GlcNAc; M*x* indicates the number of mannose residues on core GlcNAcs; A*x* indicates the number of antenna (GlcNAc) on the trimannosyl core: A2, biantennary with both GlcNAcs β1,2-linked; A3, triantennary with a GlcNAc linked β1,2 to both mannose residues and the third GlcNAc linked β1,4 to the α1,3-linked mannose; B indicates bisecting GlcNAc linked β1,4 to β1,3-mannose; G*x* indicates the number of β1,4-linked galactose residues on the antenna; S*x* indicates the number of sialic acids linked to galactose

**Table 3:** Associations of the directly measured and derived IgG *N*-glycans with disease status, further adjusted for age and sex, and corrected for multiple testing

Glycan	Description^a^	OR	95% CI	SE	*p* value
GP5	M5 glycan	1.53	1.17, 1.99	0.21	3.89 × 10^−3^
GP6	FA2B glycan	1.71	1.31, 2.24	0.23	3.02 × 10^−4^
GP8	FA2[6]G1 glycan	0.40	0.30, 0.54	0.06	7.95 × 10^−9^
GP9	FA2[3]G1 glycan	0.69	0.52, 0.93	0.10	2.73 × 10^−2^
GP11	FA2[3]BG1 glycan	1.90	1.43, 2.54	0.28	4.26 × 10^−5^
GP15	FA2BG2 glycan	2.09	1.52, 2.87	0.34	3.49 × 10^−5^
GP17	A2G2S1 glycan	1.41	1.07, 1.85	0.20	2.73 × 10^−2^
GP19	FA2BG2S1 glycan	2.91	2.09, 4.05	0.49	4.90 × 10^−9^
GP20	Structure not determined	1.60	1.21, 2.10	0.22	2.29 × 10^−3^
GP21	A2G2S2 glycan	1.69	1.28, 2.24	0.24	6.24 × 10^−4^
GP22	A2BG2S2 glycan	1.39	1.07, 1.81	0.19	2.73 × 10^−2^
GP24	FA2BG2S2 glycan	2.62	1.94, 3.54	0.40	4.90 × 10^−9^
B	Structures with bisecting GlcNAc	2.16	1.61, 2.89	0.32	1.65 × 10^−6^
G1	Monogalactosylated structures	0.37	0.27, 0.51	0.06	7.95 × 10^−9^
HM	High-mannose structures	1.53	1.17, 1.99	0.21	3.89 × 10^−3^
S0	Asialylated structures	0.50	0.37, 0.68	0.08	3.56 × 10^−5^
S2	Disialylated structures	1.86	1.41, 2.45	0.26	4.19 × 10^−5^

Only statistically significant associations are presented; for all associations see ESM Table 10

^a^The description relates to the percentage of that glycan in total IgG *N*-glycans

GP, glycan peak. Structure abbreviations: all *N*-glycans have two core GlcNAcs; F at the start of the abbreviation indicates a core fucose α1,6-linked to the inner GlcNAc; M*x* indicates the number of mannose residues on core GlcNAcs; A*x* indicates the number of antenna (GlcNAc) on the trimannosyl core: A2, biantennary with both GlcNAcs β1,2-linked; B indicates bisecting GlcNAc linked β1,4 to β1,3-mannose; G*x* indicates the number of β1,4-linked galactose residues on the antenna; S*x* indicates the number of sialic acids linked to galactose

### Decreased plasma and IgG monogalactosylation in type 1 diabetes

There was a significant decrease in the proportion of plasma and IgG monogalactosylated *N-*glycans (G1 derived trait) in children with type 1 diabetes. Considering all IgG derived traits, the most significant difference between studied groups was observed for the G1 trait (OR = 0.37, *p*=7.95 × 10^−9^).

### Increased plasma and IgG bisecting GlcNAc in type 1 diabetes

The derived trait of total bisecting GlcNAc was increased in children with type 1 diabetes when compared with their healthy siblings, in both plasma *N-*glycans (OR = 1.72, *p*=3.52 × 10^−3^) and IgG *N-*glycans (OR = 2.16, *p*=1.65 × 10^−6^). Among plasma *N-*glycans, the most significant difference between studied groups was observed for the GP2 *N-*glycan with bisecting GlcNAc (FA2B) (OR = 2.09, *p*=1.16 × 10^−5^). Both plasma and IgG FA2B proportions were increased in children with type 1 diabetes.

### Increased IgG disialylation in type 1 diabetes

IgG asialylated glycans were significantly decreased in children with type 1 diabetes compared with their healthy siblings (OR = 0.50, *p*=3.56 × 10^−5^), whereas IgG disialylated glycans were increased (OR = 1.86, *p*=4.19 × 10^−5^). This was mainly driven by an increase in FA2BG2S2/GP24 (OR = 2.62, *p*=4.90 × 10^−9^). Among directly measured plasma *N-*glycans, significant increases were observed for some of the monosialylated, disialylated and trisialylated structures in children with type 1 diabetes relative to their healthy siblings. The most significant increase was observed for the disialylated A3G3S2 (GP25) glycan (OR = 2.12, *p*=4.54 × 10^−5^).

### Increased plasma and IgG high-mannose glycans in type 1 diabetes

IgG high-mannose glycans (GP5) were significantly increased in children with type 1 diabetes relative to their healthy siblings (OR = 1.53, *p*=3.89 × 10^−3^). Among plasma proteins, the derived trait describing high-mannose structures differed most significantly among all tested derived traits between studied groups (OR = 1.65, *p*=2.16 × 10^−3^). This is driven by the increase in GP7 and GP12 (indicating mannose glycans with six and seven mannose subunits, respectively) among children with type 1 diabetes relative to the healthy siblings.

### Association of plasma and IgG *N-*glycan proportions with levels and number of specific autoantibodies

Next, we examined the association of plasma and IgG *N-*glycan proportions with levels and number of specific autoantibodies (ESM Figs 5, 6, 7 and 8). The IgG GP13 *N-*glycan was significantly associated (*p*=7.66 × 10^−5^, *β* = 0.590) with ZnT8RA levels.

We found that increased number of autoantibodies (1–4) was accompanied by a significant decrease in the proportion of some of the highly branched plasma *N-*glycans (ESM Table 8). The most significant decrease was observed for GP30, representing trigalactosylated and trisialylated triantennary *N-*glycan (*p*=4.04 × 10^−4^, *β* = −0.931).

### *N-*glycan proportions differed between sex, and correlated with age at diagnosis of type 1 diabetes

**Fig. 2:**
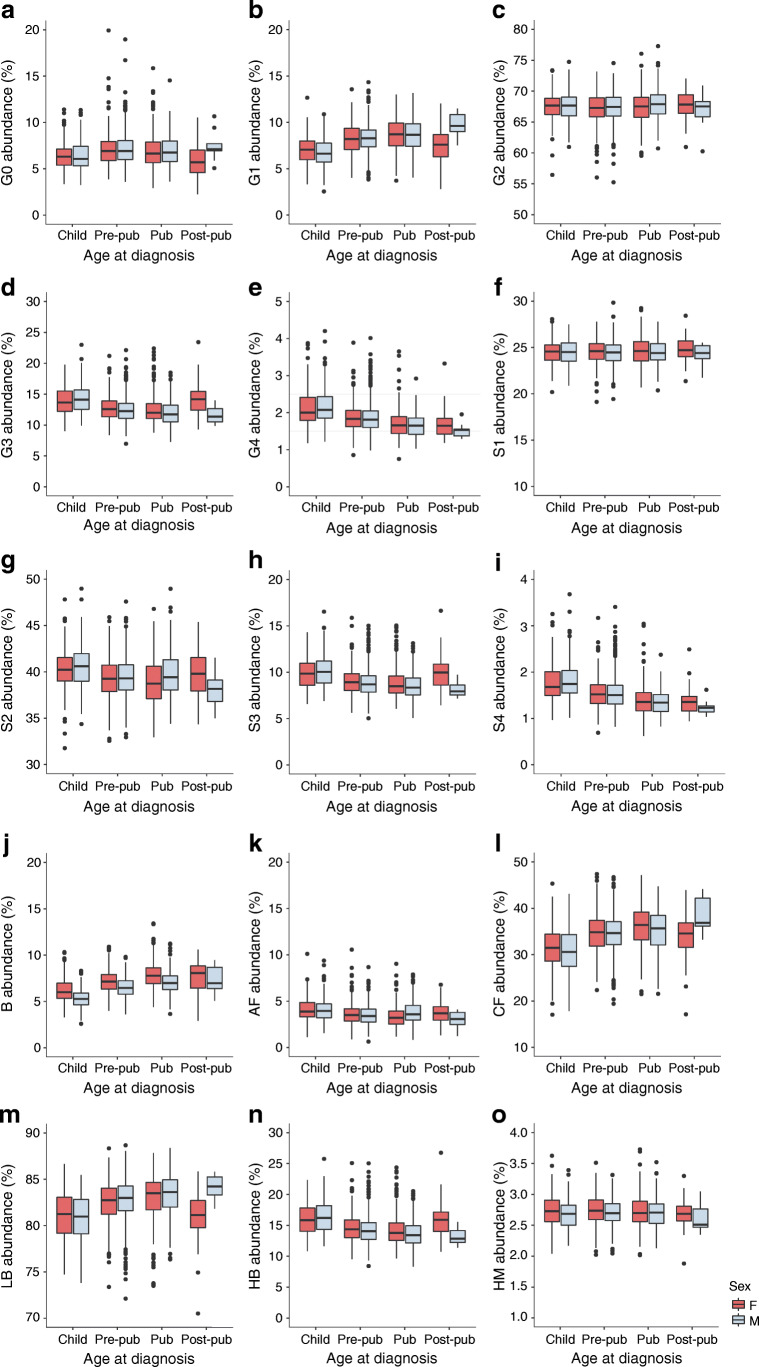
Proportions of derived plasma *N*-glycans in groups comprising male and female children with various ages at diagnosis of type 1 diabetes. Child, age 0.6–6 years; Pre-pub, pre-pubertal children, age 6–11/13 years; Pub, pubertal children, age 11/13–15/16.9 years; Post-pub, post-pubertal children, age 15/17–19.1/18.3 years; F, female; M, male; G0, agalactosylated glycans; G1, monogalactosylated glycans; G2, digalactosylated glycans; G3, trigalactosylated glycans; G4, tetragalactosylated glycans; S1, monosialylated glycans; S2, disialylated glycans; S3, trisialylated glycans; S4, tetrasialylated glycans; B, glycans with bisecting GlcNAc; AF, glycans with antennary fucose; CF, glycans with core fucose; LB, low branching glycans; HB, high branching glycans; HM, high-mannose glycans

**Fig. 3:**
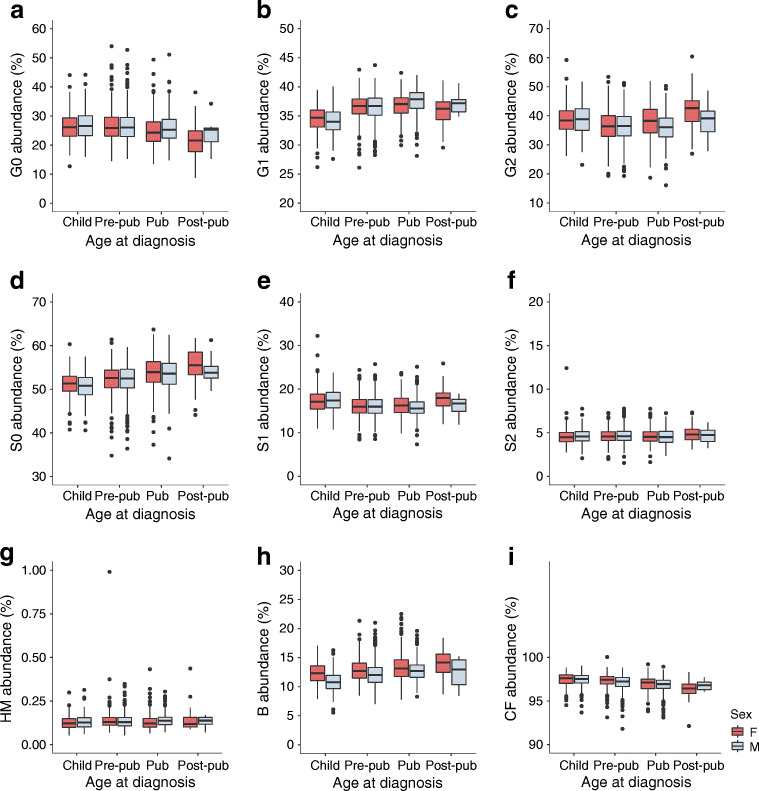
Proportions of derived IgG *N*-glycans in groups comprising male and female children with various ages at diagnosis of type 1 diabetes. See Fig. 2 legend for definition of age categories and glycans

The primary study population, comprising 1917 children with type 1 diabetes, was divided based on sex and over four age categories (Table 1). We were particularly interested in sex differences and correlations with age at diagnosis for those plasma and IgG *N-*glycans that significantly differed between healthy and diseased siblings, and focused on the derived glycan traits shown in Figs 2 and 3. Proportions of directly measured glycans in groups comprising male and female children with various ages at diagnosis of type 1 diabetes are presented in ESM Figs 9 and 10.

Differences in the proportions of monogalactosylated (G1) glycans were detected in the plasma and IgG fractions. Overall, in male participants, G1 proportions were higher at the onset of puberty and increased with age at diagnosis. Plasma G1 proportions decreased in the post-pubertal group in female participants, whereas an increase over time was observed in other age groups. Both plasma and IgG proportions of bisecting GlcNAc (B) differed between sexes, and were generally lower in male than in female participants in all age categories. In both sexes, there was an overall increase in B proportions with age at diagnosis. The proportions of high-mannose glycans (HM) in plasma were higher in the very young and pre-pubertal female participants, whereas IgG HM glycans were higher in pubertal male participants. The proportion of IgG HM glycans in female participants increased in the pre-pubertal age group, then decreased at puberty. In male participants, the proportion of IgG HM glycans increased in the pubertal age group in comparison with very young children. An overall increase with age at diagnosis was observed for IgG asialylation (S0) in both sexes.

### Discriminating children with type 1 diabetes from their healthy siblings based on plasma and IgG *N-*glycan profiles

**Fig. 4:**
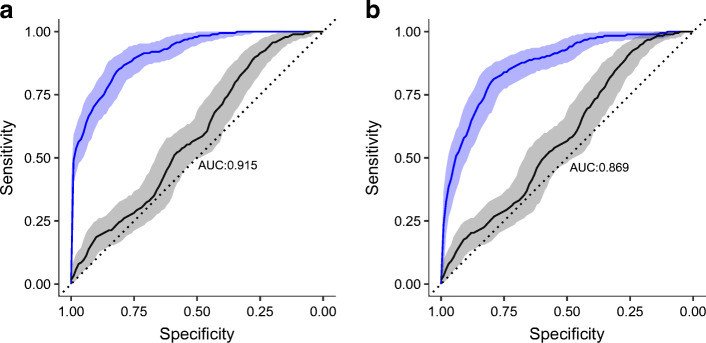
Receiver operating characteristic curves showing the performance of a glycan-based discriminative model in predicting disease status of patients with type 1 diabetes and their healthy siblings. Models based on age and sex did not show a discriminative power (black lines), while addition of plasma (**a**) and IgG (**b**) *N*-glycan traits increased the discriminative power of the model (blue lines)

A glycan-based discriminative model was built using logistic mixed-model elastic net regression. Only directly measured *N-*glycans (24 IgG *N-*glycans or 39 plasma *N-*glycans) were used as predictors. To evaluate model performance, a 10-fold cross-validation was used. A model based only on age and sex did not have significant discriminative power (AUC 0.584). Addition of *N-*glycans into the model increased the discriminative power for both IgG *N-*glycans (AUC 0.869; 95% CI 0.833, 0.900) and plasma *N-*glycans (AUC 0.915; 95% CI 0.888, 0.939) (Fig. 4). Classification performance of individual *N-*glycans identified by receiver operating characteristic curve analyses is presented in ESM Fig. 11.

## Discussion

In the first part of this study, plasma and IgG *N-*glycans were analysed in a study population comprising 1917 children and adolescents (age 0.6–19.1 years) who were newly diagnosed with type 1 diabetes. A follow-up study compared the results for 188 of these participants with those for 244 unaffected siblings. As far as we are aware, these are the largest studies of plasma *N-*glycosylation changes in children to date, and the first to be performed at the onset of type 1 diabetes. We found significant differences in both plasma and IgG *N-*glycans between children with type 1 diabetes and their healthy siblings, and developed a glycan-based type 1 diabetes discriminative model. We also found significant *N-*glycan changes with age at diagnosis of type 1 diabetes and between sexes in diabetic patients.

Our previous study of plasma and IgG *N-*glycosylation in adult type 1 diabetes patients showed that higher HbA_1c_ was associated with a shift toward more complex triantennary and tetraantennary plasma *N-*glycans [22]. In the current study, we observed a different change at the onset of type 1 diabetes, toward simpler *N-*glycans, i.e. very simple plasma and IgG glycans with terminal mannoses and GlcNAcs, some biantennary plasma *N-*glycans, and *N-*glycans with bisecting GlcNAc. These changes specific to individuals with recent onset of diabetes suggest that they may have value in prediction of type 1 diabetes risk, and may not be merely the reflection of differences in glycaemic control.

A significant increase in the proportion of glycans that terminate with mannoses and glycans with bisecting GlcNAc was observed in children with type 1 diabetes in comparison with their healthy siblings. An increase in the mannose-binding lectin that binds to terminal mannoses and GlcNAcs has been reported in populations of individuals with type 1 diabetes [24, 39]. In rheumatoid arthritis, multiple presentation of IgG *N-*glycans with terminal GlcNAcs was shown to activate the mannose-binding lectin-complement cascade in the affected joints [40], suggesting that these glycan changes may contribute to the chronic joint inflammation. In the present study, both plasma and IgG FA2B glycan (which terminates in three GlcNAcs) increased in the type 1 diabetes group. Also, the gene encoding the main protein of the complement activation pathway, complement C3 protein, has been associated with an increased risk of type 1 diabetes development among HLA-DR4/4 carriers [41], further demonstrating an important role of the complement system in type 1 diabetes. IgGs carrying bisecting GlcNAc have been implicated in increased antibody-dependent cellular cytotoxicity [42], an important process during virus elimination, and it has been suggested that one of the autoimmunity triggers in type 1 diabetes may be virus-derived [43].

In addition, we observed an increase in IgG disialylation and a decrease in asialylation proportion among children with type 1 diabetes. Studies have shown that sialylated IgGs are anti-inflammatory mediators [44]. We speculate that differences in sialylation between the studied groups may reflect the ongoing inflammation process at the onset of this disease.

A decrease in monogalactosylation proportion was also observed in children with type 1 diabetes relative to their healthy siblings. This has also been reported previously in another autoimmune disease, systemic lupus erythematosus [45]. However, a decrease in the proportion of FA2[3]G1 and FA2[6]G1 monogalactosylated glycans was associated with poorer glycaemic control in adult type 1 diabetes patients [22], and thus its real individual value in risk assessment should be evaluated after correcting for glycaemic differences.

Significant *N-*glycosylation differences between the sexes were mainly observed upon onset of puberty, which is in line with our previous study of 170 children and adolescents [46]. However, derived traits representing plasma and IgG bisecting structures, plasma high-mannose structures and IgG core fucose were significantly different between the sexes even before puberty. It is reasonable to examine sex differences for disease-associated glycans as there may be a hormonal component associated with type 1 diabetes given the higher prevalence of this disease in male participants after the onset of puberty [47]. However, the proportions of some of the disease-associated risk-increasing glycans were higher and those of the disease-associated risk-decreasing glycans were lower in the same sex. Therefore, it is difficult to speculate which glycans reflect the different risk rates between the sexes. A previous study of 130 children and adolescents [48] reported similar overall changes of IgG glycosylation with age.

ZnT8R autoantibodies (ZnT8RA) were associated with the digalactosylated IgG *N-*glycan with bisecting GlcNAc (GP13), and an increase in the number of different autoantibodies, which is thought to be a better predictor of progression to type 1 diabetes than levels of any individual antibody [3], was associated with some of the highly branched plasma *N-*glycans. These results indicate that some variations in glycans reflect type 1 diabetes-specific autoimmunity. ZnT8A were detected in 81% of children who progressed to type 1 diabetes [49], making these autoantibodies very important in predicting diabetes. However, the proportion of GP13 glycan was not significantly different between affected and unaffected siblings. In the present study, we have not distinguished between the fraction of IgG antibodies that react to type 1 diabetes antigens and the non-autoreactive IgG fraction. Studying glycosylation changes of type 1 diabetes antigen-specific IgGs would be important in future studies.

As the plasma samples analysed in the current study were collected within 3 months of type 1 diabetes onset, the study design does not allow us to conclude whether the observed *N-*glycosylation changes are causative or reflective of disease status. We also acknowledge that, when studying total plasma protein *N-*glycome, both variation in protein glycosylation and changes in protein concentration could affect the observed plasma glycome changes. However, *N-*glycosylation appears to be strongly associated with type 1 diabetes, and this association demands further research. We were not able to perform a validation study as it is very hard to obtain samples from patients at the onset of this disease; thus, evaluation of the utility of glycan-based predictive model remains an important future step. Also, we defined puberty based on age categories, whereas clinical assessment would be a more precise measure. We were not able to standardise the glycan data against medication intake as data regarding the treatment of study participants were not available. Nevertheless, our previous study demonstrated that insulin intake has a low effect on a limited number of glycans [50]. Some of the unaffected siblings were lost to follow-up if they were subsequently diagnosed above the age of 18 years, and therefore their type 1 diabetes status is less certain than for those individuals who were followed for an extended time. However, as the incidence of type 1 diabetes after puberty decreases markedly with increasing age [47], it is less likely that the older individuals followed for a shorter period developed the disease. One of the major strengths of our study is that the study population comprises children at the onset of type 1 diabetes together with their unaffected siblings, without other comorbidities as seen in the adult population, allowing glycan changes related to type 1 diabetes to be investigated more precisely.

Earlier intervention to prevent type 1 diabetes would be aided by identifying individuals who are at higher risk, hence the long-running search for novel biomarkers of this disease. The main contribution of our research study was the identification of *N-*glycosylation changes around the time of diabetes onset. This allowed us to develop a glycan-based predictive model that may be of clinical utility (AUC >0.9). This model outperforms a previously described genetic risk score [51], which, when combined with additional clinical data, yielded an AUC of 0.79, and compares favourably with the recently improved version of this score [6]. Incorporation of genetic and clinical data into the differential *N-*glycosylation model could further optimise prediction. An important future step to evaluate the utility of the glycan-based predictive model would be to study whether the *N-*glycosylation profile identified in children at type 1 diabetes onset also exists in individuals who have islet autoimmunity before the development of clinical diabetes. Interestingly, two unaffected siblings who had very low monogalactosylation proportions (below Q1) at the time of plasma collection were later diagnosed with diabetes. This glycan trait was significantly lower in children with type 1 diabetes in comparison with their healthy siblings.

In summary, the current study demonstrated significant changes in plasma *N-*glycosylation accompanying the onset of type 1 diabetes, and enabled us to develop a predictive model incorporating glycan data. Our large cohort also made it possible to confirm age- and sex-dependent *N-*glycosylation changes in children and adolescents studied to date on a smaller number of participants, and to reveal some new differences. An increase in the number of different type 1 diabetes-associated autoantibodies, which is a better predictor of progression to diabetes than any individual antibody, was associated with specific changes within the plasma *N-*glycome. These results favour further research into *N-*glycosylation changes and their impact on type 1 diabetes.

## Supplementary information

ESM 1 (PDF 2.39 mb)

## Data Availability

The datasets generated during and/or analysed during the current study are available from the corresponding author on reasonable request.

## Abbreviations

HILIC-UPLC: Hydrophilic interaction ultra-performance liquid chromatography
IA-2: Insulinoma-associated protein 2
UDP-GlcNAc: Uridine diphosphate-*N-*acetylglucosamine
ZnT8R: Arginine zinc transporter 8
ZnT8W: Tryptophan zinc transporter 8
