# Supplementary material for: Children at onset of type 1 diabetes show altered N-glycosylation of plasma proteins and IgG
Source: Diabetologia. 2022 May 27;65(8):1315–27. doi: 10.1007/s00125-022-05703-8 (PMC9283363; doi:10.1007/s00125-022-05703-8)
Supplement: Supplementary file 1 — (PDF 2.39 mb) [file 125_2022_5703_MOESM1_ESM.pdf]

ESM Table 1 General description of the IgG glycan peaks

Glycan peak	Glycan structure[a]	Glycan description[b]
GP1	FA1	core fucosylated, monantennary
GP2	A2	biantennary
GP3	A2B	biantennary with bisecting GlcNAc
GP4	FA2	core fucosylated, biantennary
GP5	M5<br>FA2	high-mannose<br>core fucosylated, biantennary
GP6	FA2B	core fucosylated, biantennary with bisecting GlcNAc
GP7	A2[3]G1<br>FA2B	monogalactosylated, biantennary<br>core fucosylated, biantennary with bisecting GlcNAc
GP8	FA2[6]G1	core fucosylated, monogalactosylated, biantennary
GP9	FA2[3]G1	core fucosylated, monogalactosylated, biantennary
GP10	FA2[6]BG1	core fucosylated, monogalactosylated, biantennary with bisecting GlcNAc
GP11	FA2[3]BG1	core fucosylated, monogalactosylated, biantennary with bisecting GlcNAc
GP12	A2G2	digalactosylated, biantennary
GP13	A2BG2	digalactosylated, biantennary with bisecting GlcNAc
GP14	FA2G2	core fucosylated, digalactosylated, biantennary
GP15	FA2BG2	core fucosylated, digalactosylated, biantennary with bisecting GlcNAc
GP16	FA2[3]G1S1	core fucosylated, monogalactosylated and monosialylated, biantennary
GP17	A2G2S1	digalactosylated and monosialylated, biantennary
GP18	FA2G2S1	core fucosylated, digalactosylated and monosialylated, biantennary
GP19	FA2BG2S1	core fucosylated, digalactosylated and monosialylated, biantennary with bisecting GlcNAc
GP20	not determined	
GP21	A2G2S2	digalactosylated and disialylated, biantennary
GP22	A2BG2S2	digalactosylated and disialylated, biantennary with bisecting GlcNAc
GP23	FA2G2S2	core fucosylated, digalactosylated and disialylated, biantennary
GP24	FA2BG2S2	core fucosylated, digalactosylated and disialylated, biantennary with bisecting GlcNAc

[a]The most abundant glycan structure in glycan peak
[b]The description relates to the percentage of that glycan in total IgG *N*-glycans.
Structure abbreviations: all *N*-glycans have two core GlcNAcs; F at the start of the abbreviation indicates a core fucose α1,6-linked to the inner GlcNAc; Mx indicates the number of mannose residues on core GlcNAcs; Ax indicates the number of antenna (GlcNAc) on the trimannosyl core; A2, biantennary with both GlcNAcs as β1,2-linked; B, bisecting GlcNAc linked β1,4 to β1,3-mannose; Gx indicates the number of β1,4-linked galactose residues on the antenna; Sx indicates the number of sialic acids linked to galactose. (Pučić et al, Mol. Cell Proteomics, 2011, 10, M111.010090)

ESM Table 2 General description of the plasma glycan peaks

**Glycan peak**	**Glycan structure[a]**	**Glycan description[b]**
GP1	FA2	core fucosylated, biantennary
GP2	FA2B	core fucosylated, biantennary with bisecting GlcNAc
GP3	A2BG1	monogalactosylated, biantennary with bisecting GlcNAc
GP4	FA2[6]G1	core fucosylated and monogalactosylated, biantennary
GP5	FA2[3]G1	core fucosylated and monogalactosylated, biantennary
GP6	FA2[6]BG1	core fucosylated and monogalactosylated, biantennary with bisecting GlcNAc
GP7	M6	high-mannose
GP8	A2G2	digalactosylated, biantennary
GP9	A2BG2	digalactosylated, biantennary with bisecting GlcNAc
GP10	FA2G2	core fucosylated, digalactosylated, biantennary
GP11	FA2BG2	core fucosylated, digalactosylated, biantennary with bisecting GlcNAc
GP12	M7<br>A2G2S1	high-mannose<br>digalactosylated, monosialylated, biantennary
GP13	FA2G1S1	core fucosylated, monogalactosylated and monosialylated biantennary
GP14	A2G2S1	digalactosylated and monosialylated biantennary
GP15	A2BG2S1	digalactosylated and monosialylated biantennary with bisecting GlcNAc
GP16	FA2G2S1	core fucosylated, digalactosylated and monosialylated biantennary
GP17	FA2BG2S1	core fucosylated, digalactosylated and monosialylated biantennary with bisecting GlcNAc
GP18	A2G2S2	digalactosylated and disialylated biantennary
GP19	M9	high-mannose
GP20	A2G2S2	digalactosylated and disialylated biantennary
GP21	A2G2S2	digalactosylated and disialylated biantennary

GP22	FA2G2S2	core fucosylated, digalactosylated and disialylated biantennary
GP23	FA2BG2S2	core fucosylated, digalactosylated and disialylated biantennary with bisecting GlcNAc
GP24	A3G3S2	trigalactosylated and disialylated triantennary
GP25	A3G3S2	trigalactosylated and disialylated triantennary
GP26	A3G3S2	trigalactosylated and disialylated triantennary
GP27	A3F1G3S2	antennary fucosylated, trigalactosylated and disialylated, triantennary
GP28	A3G3S3	trigalactosylated and trisialylated, triantennary
GP29	A3G3S3	trigalactosylated and trisialylated, triantennary
GP30	A3G3S3	trigalactosylated and trisialylated, triantennary
GP31	FA3G3S3	core fucosylated, trigalactosylated and trisialylated triantennary
GP32	A3G3S3	trigalactosylated and trisialylated, triantennary
GP33	A3F1G3S3	antennary fucosylated, trigalactosylated and trisialylated, triantennary
GP34	FA3G3S3	core fucosylated, trigalactosylated and trisialylated, triantennary
GP35	FA3F1G3S3	core and antennary fucosylated, trigalactosylated and trisialylated, triantennary
GP36	A4G4S3	tetragalactosylated and trisialylated, tetraantennary
GP37	A4G4S4	tetragalactosylated and tetrasialylated tetraantennary
GP38	A4G4S4	tetragalactosylated and tetrasialylated tetraantennary
GP39	A4F1G4S4	antennary fucosylated, tetragalactosylated and tetrasialylated tetraantennary

[a]The most abundant glycan structure in glycan peak
[b]The description relates to the percentage of that glycan in total plasma *N*-glycans.
Structure abbreviations: all *N*-glycans have two core GlcNAcs; F at the start of the abbreviation indicates a core fucose α1,6-linked to the inner GlcNAc; Mx indicates the number of mannose residues on core GlcNAcs; Ax indicates the number of antenna (GlcNAc) on the trimannosyl core: A2, biantennary with both GlcNAcs as β1,2-linked; A3, triantennary with a GlcNAc linked β1,2 to both mannose residues and the third GlcNAc linked β1,4 to the α1,3-linked mannose; A4, GlcNAcs linked as A3 with the additional GlcNAc β1,6-linked to α1,6-mannose; B indicates bisecting GlcNAc linked β1,4 to β1,3-mannose; Gx indicates the number of β1,4-linked galactose residues on the antenna; Fx indicates the number of fucose linked α1,3 to the antenna GlcNAc; Sx indicates the number of sialic acids linked to galactose. (Zaytseva et al, J Proteome Res, 2020)

ESM Table 3 Calculation of the IgG derived traits

**Glycan peak**	**G0**	**G1**	**G2**	**S0**	**S1**	**S2**	**B**	**CF**	**HM**
GP1	1	0	0	0	0	0	0	1	0

GP2	1	0	0	0	0	0	0	0	0
GP3	0	0	0	0	0	0	0	0	0
GP4	1	0	0	0	0	0	0	1	0
GP5	0	0	0	0	0	0	0	0	1
GP6	1	0	0	0	0	0	1	1	0
GP7	0	1	0	1	0	0	0	0	0
GP8	0	1	0	1	0	0	0	1	0
GP9	0	1	0	1	0	0	0	1	0
GP10	0	1	0	1	0	0	1	1	0
GP11	0	1	0	1	0	0	1	1	0
GP12	0	0	1	1	0	0	0	0	0
GP13	0	0	1	1	0	0	1	0	0
GP14	0	0	1	1	0	0	0	1	0
GP15	0	0	1	1	0	0	1	1	0
GP16	0	1	0	0	1	0	0	1	0
GP17	0	0	1	0	1	0	0	0	0
GP18	0	0	1	0	1	0	0	1	0
GP19	0	0	1	0	1	0	1	1	0
GP20	0	0	0	0	0	0	0	0	0
GP21	0	0	1	0	0	1	0	0	0
GP22	0	0	1	0	0	1	1	0	0
GP23	0	0	1	0	0	1	0	1	0
GP24	0	0	1	0	0	1	1	1	0

G0, agalactosylated glycans; G1, monogalactosylated glycans; G2, digalactosylated glycans; S0, asialylated glycans; S1, monosialylated glycans; S2, disialylated glycans; B, glycans with bisecting *N*-acetylglucosamine (GlcNAc); CF, glycans with core fucose; HM, high-mannose glycans; 0, glycan peak (GP) area is not included in the calculation; 1, GP area is included in total in the calculation

ESM Table 4 Calculation of the plasma derived traits

**Glycan peak**	**LB**	**HB**	**G0**	**G1**	**G2**	**G3**	**G4**	**S1**	**S2**	**S3**	**S4**	**B**	**AF**	**CF**	**HM**
GP1	1	0	1	0	0	0	0	0	0	0	0	0	0	1	0
GP2	1	0	1	0	0	0	0	0	0	0	0	1	0	1	0
GP3	1	0	0	1	0	0	0	0	0	0	0	1	0	0	0
GP4	1	0	0	1	0	0	0	0	0	0	0	0	0	1	0
GP5	1	0	0	1	0	0	0	0	0	0	0	0	0	1	0
GP6	1	0	0	1	0	0	0	0	0	0	0	1	0	1	0
GP7	0	0	0	0	0	0	0	0	0	0	0	0	0	0	1
GP8	1	0	0	0	1	0	0	0	0	0	0	0	0	0	0
GP9	1	0	0	0	1	0	0	0	0	0	0	1	0	0	0
GP10	1	0	0	0	1	0	0	0	0	0	0	0	0	1	0
GP11	1	0	0	0	1	0	0	0	0	0	0	1	0	1	0

GP12	0.5	0	0	0	0.5	0	0	0.5	0	0	0	0	0	0	0.5
GP13	1	0	0	1	0	0	0	1	0	0	0	0	0	1	0
GP14	1	0	0	0	1	0	0	1	0	0	0	0	0	0	0
GP15	1	0	0	0	1	0	0	1	0	0	0	1	0	0	0
GP16	1	0	0	0	1	0	0	1	0	0	0	0	0	1	0
GP17	1	0	0	0	1	0	0	1	0	0	0	1	0	1	0
GP18	1	0	0	0	1	0	0	0	1	0	0	0	0	0	0
GP19	0	0	0	0	0	0	0	0	0	0	0	0	0	0	1
GP20	1	0	0	0	1	0	0	0	1	0	0	0	0	0	0
GP21	1	0	0	0	1	0	0	0	1	0	0	0	0	0	0
GP22	1	0	0	0	1	0	0	0	1	0	0	0	0	1	0
GP23	1	0	0	0	1	0	0	0	1	0	0	1	0	1	0
GP24	0	1	0	0	0	1	0	0	1	0	0	0	0	0	0
GP25	0	1	0	0	0	1	0	0	1	0	0	0	0	0	0
GP26	0	1	0	0	0	1	0	0	1	0	0	0	0	0	0
GP27	0	1	0	0	0	1	0	0	1	0	0	0	1	0	0
GP28	0	1	0	0	0	1	0	0	0	1	0	0	0	0	0
GP29	0	1	0	0	0	1	0	0	0	1	0	0	0	0	0
GP30	0	1	0	0	0	1	0	0	0	1	0	0	0	0	0
GP31	0	1	0	0	0	1	0	0	0	1	0	0	0	1	0
GP32	0	1	0	0	0	1	0	0	0	1	0	0	0	0	0
GP33	0	1	0	0	0	1	0	0	0	1	0	0	1	0	0
GP34	0	1	0	0	0	1	0	0	0	1	0	0	0	1	0
GP35	0	1	0	0	0	1	0	0	0	1	0	0	1	1	0
GP36	0	1	0	0	0	0	1	0	0	1	0	0	0	0	0
GP37	0	1	0	0	0	0	1	0	0	0	1	0	0	0	0
GP38	0	1	0	0	0	0	1	0	0	0	1	0	0	0	0
GP39	0	1	0	0	0	0	1	0	0	0	1	0	1	0	0

LB*,* low branched glycans; HB*,* highly branched glycans; G0*,* agalactosylated glycans; G1*,* monogalactosylated glycans; G2*,* digalactosylated glycans; G3*,* trigalactosylated glycans; G4*,* tetragalactosylated glycans; S1*,* monosialylated glycans; S2*,* disialylated glycans; S3*,* trisialylated glycans; S4*,* tetrasialylated glycans; B*,* glycans with bisecting *N*-acetylglucosamine (GlcNAc); AF*,* glycans with antennary fucose; CF*,* glycans with core fucose; HM*,* high-mannose glycans; 0*,* glycan peak (GP) area is not included in the calculation; 0.5*,* half of the GP area is included in the calculation; 1*,* GP area is included in total in the calculation

ESM Table 5 Descriptive statistics of plasma and IgG *N*-glycans quantified in children with type 1 diabetes in the primary study

**Glycan peak**	**Q1**	**Median**	**Q3**
Plasma *N*-glycans			
GP1	3.85	4.64	5.7
GP2	1.75	1.96	2.21

GP3	0.03	0.04	0.05
GP4	3.95	4.69	5.38
GP5	1.5	1.86	2.21
GP6	0.84	0.99	1.17
GP7	1.02	1.1	1.2
GP8	1.76	1.94	2.11
GP9	0.06	0.08	0.09
GP10	4.5	5.1	5.77
GP11	0.61	0.73	0.87
GP12	1.48	1.61	1.75
GP13	0.37	0.44	0.51
GP14	14.49	15.36	16.23
GP15	0.19	0.22	0.25
GP16	5.61	6.16	6.79
GP17	1.08	1.38	1.75
GP18	3.26	3.65	4.07
GP19	0.72	0.78	0.84
GP20	24.88	26.25	27.7
GP21	0.39	0.43	0.46
GP22	3.24	3.73	4.28
GP23	0.95	1.18	1.47
GP24	1.48	1.72	2
GP25	0.06	0.07	0.09
GP26	1.2	1.41	1.64
GP27	0.55	0.72	0.92
GP28	0.45	0.53	0.62
GP29	0.05	0.07	0.09
GP30	3.48	4.02	4.72
GP31	0.23	0.29	0.37
GP32	0.83	1	1.19
GP33	1.77	2.19	2.67
GP34	0.21	0.25	0.29
GP35	0.11	0.15	0.2
GP36	0.27	0.31	0.36
GP37	0.28	0.36	0.45
GP38	0.58	0.68	0.8
GP39	0.36	0.44	0.54
AF	2.84	3.52	4.31
B	5.81	6.7	7.72
CF	31.22	34.39	37.43
G0	5.73	6.65	7.83
G1	6.93	8.09	9.21
G2	66.82	68.36	69.87

G3	11.26	12.55	14.08
G4	1.57	1.81	2.08
HB	12.93	14.31	16.05
HM	3.31	3.51	3.73
LB	81.81	83.57	84.97
S1	24.38	25.32	26.24
S2	38	39.51	41.04
S3	7.99	8.87	10.02
S4	1.28	1.5	1.73
IgG *N*-glycans			
GP1	0.07	0.08	0.1
GP2	0.15	0.22	0.34
GP3	0.08	0.1	0.11
GP4	18.63	21.54	24.53
GP5	0.11	0.13	0.15
GP6	3.14	3.72	4.37
GP7	0.11	0.16	0.23
GP8	19.8	20.96	22.13
GP9	7.53	8.43	9.34
GP10	3.06	3.56	4.11
GP11	0.35	0.41	0.47
GP12	0.2	0.31	0.48
GP13	0.13	0.15	0.17
GP14	15.09	16.99	18.85
GP15	1.05	1.22	1.44
GP16	2.3	2.55	2.81
GP17	0.61	0.69	0.78
GP18	9.99	11.47	13.04
GP19	1.28	1.47	1.71
GP20	0.18	0.21	0.24
GP21	0.49	0.55	0.61
GP22	0.07	0.08	0.1
GP23	2.04	2.38	2.77
GP24	1.31	1.52	1.77
B	11	12.33	13.78
CF	96.71	97.26	97.73
G0	22.36	25.66	29.08
G1	34.75	36.39	37.92
G2	33.65	37.23	40.93
S0	50.28	52.57	54.83
S1	14.74	16.24	17.93
S2	4.06	4.57	5.15

AF, glycans with antennary fucose; B, glycans with bisecting *N*-acetylglucosamine (GlcNAc); CF, glycans with core fucose; G0, agalactosylated glycans; G1, monogalactosylated glycans; G2, digalactosylated glycans; G3, trigalactosylated glycans; G4, tetragalactosylated glycans; HB, highly branched glycans; HM, high-mannose glycans; LB, low branched glycans; S1, monosialylated glycans; S2, disialylated glycans; S3, trisialylated glycans; S4, tetrasialylated glycans

ESM Table 6 Descriptive statistics of plasma *N*-glycans quantified in the follow-up family-based study

Control						Ctrl$_1$	Ctrl$_2$	Cases				
**GP**	**Med**	**Q1**	**Q3**	**Min**	**Max**	**Area**	**Area**	**Med**	**Q1**	**Q3**	**Min**	**Max**
GP1	4.53	3.66	5.36	1.5	11.59	3.89	3.06	4.62	3.71	5.81	2.02	9.6
GP2	1.74	1.58	1.94	1.03	3.48	1.62	1.03	1.92	1.71	2.17	1.19	3
GP3	0.05	0.04	0.07	0.03	0.13	0.07	0.03	0.05	0.04	0.06	0.02	0.12
GP4	5.02	4.46	5.57	2.62	8.35	3.67	3.33	4.65	3.86	5.21	2.46	7.19
GP5	2.05	1.79	2.35	0.85	3.53	1.24	1.37	1.86	1.49	2.21	0.81	3.29
GP6	1.06	0.92	1.24	0.47	2.31	0.78	0.47	1.01	0.87	1.18	0.55	1.84
GP7	1.05	0.94	1.15	0.59	1.54	0.79	0.73	1.08	0.99	1.19	0.79	1.57
GP8	2	1.83	2.19	1.34	3	2.18	1.95	2.03	1.88	2.18	1.38	2.71
GP9	0.1	0.09	0.12	0.05	0.19	0.09	0.05	0.1	0.09	0.11	0.05	0.15
GP10	5.43	4.97	5.91	3.37	8.82	4.17	3.37	4.93	4.45	5.51	3.1	7.99
GP11	0.83	0.71	0.98	0.37	1.68	0.54	0.37	0.81	0.69	0.99	0.39	1.68
GP12	1.48	1.36	1.65	1.03	2.04	1.56	1.23	1.6	1.47	1.74	1.09	2.19
GP13	0.58	0.48	0.68	0.27	1.12	0.31	0.41	0.54	0.45	0.64	0.27	0.97
GP14	14.7	13.96	15.42	11.78	17.77	16.23	15.64	15.06	14.3	15.77	12.11	17.58
GP15	0.42	0.38	0.46	0.27	0.69	0.31	0.3	0.42	0.38	0.45	0.28	0.6
GP16	6.27	5.72	6.81	4.22	8.66	4.88	4.88	6.1	5.63	6.74	4.46	9.01
GP17	1.55	1.32	1.88	0.8	3.61	0.83	0.8	1.59	1.31	2	0.69	3.46
GP18	3.61	3.35	4.02	2.61	5.24	4.23	3.44	3.73	3.4	4.13	2.46	5.72
GP19	0.99	0.94	1.06	0.56	1.23	0.98	0.8	0.99	0.95	1.06	0.76	1.21
GP20	24.18	23.04	25.33	18.54	29.84	27.96	29.1	24.35	23.12	25.74	18.89	30.75
GP21	0.54	0.5	0.58	0.39	0.69	0.59	0.55	0.56	0.53	0.6	0.43	0.72
GP22	3.35	3.03	3.7	2.33	5.86	2.78	3.54	3.52	3.14	3.97	2.04	5.38
GP23	1.26	1.06	1.48	0.52	2.74	0.77	0.89	1.31	1.07	1.54	0.65	2.87

GP24	1.88	1.67	2.24	1.21	3.52	2.23	1.82	1.85	1.59	2.17	1.23	3.43
GP25	0.18	0.17	0.19	0.13	0.24	0.19	0.19	0.19	0.18	0.21	0.13	0.31
GP26	1.67	1.51	1.9	1.03	3	1.84	1.87	1.65	1.47	1.88	1.12	2.88
GP27	0.83	0.7	1.01	0.15	1.56	1.18	1.49	0.86	0.7	1.03	0.14	1.97
GP28	0.63	0.55	0.72	0.16	1.31	0.71	0.62	0.64	0.54	0.72	0.41	1.24
GP29	0.19	0.17	0.22	0.04	0.32	0.23	0.26	0.21	0.19	0.24	0.11	0.37
GP30	4.16	3.74	4.85	2.59	7.46	5.01	4.94	4.05	3.54	4.81	2.58	7.53
GP31	0.36	0.3	0.42	0.19	1.05	0.35	0.4	0.36	0.31	0.45	0.2	0.75
GP32	0.98	0.85	1.14	0.59	1.77	1.15	1.62	0.93	0.82	1.1	0.54	1.79
GP33	2	1.66	2.44	0.48	4.05	2.96	4.05	2.09	1.69	2.53	0.57	4.62
GP34	0.33	0.3	0.37	0.2	0.5	0.37	0.44	0.34	0.3	0.39	0.18	0.64
GP35	0.25	0.21	0.29	0.13	0.5	0.31	0.44	0.27	0.23	0.32	0.12	0.56
GP36	0.5	0.46	0.56	0.32	0.93	0.63	0.93	0.51	0.47	0.58	0.3	0.96
GP37	0.45	0.38	0.51	0.24	0.77	0.6	0.67	0.45	0.38	0.53	0.24	1.15
GP38	0.81	0.73	0.91	0.45	1.61	1.08	1.61	0.82	0.73	0.93	0.46	1.72
GP39	0.57	0.46	0.67	0.27	1.57	0.79	1.57	0.57	0.49	0.7	0.27	1.36
HB	16.09	14.77	17.77	11.31	23.2	19.62	22.91	15.7	14.47	17.94	9.84	25.27
**HM**	3.56	3.32	3.78	2.19	4.29	3.33	2.75	3.67	3.47	3.89	2.9	4.71
LB	81.77	80.13	83.08	75.41	86.7	78.7	75.82	82.02	79.87	83.36	72.6	87.28
S1	25.14	24.29	26.11	21.04	29.1	24.13	23.26	25.46	24.52	26.49	21.7	29.5
S2	37.79	36.69	39.06	31.62	42.9	41.77	42.9	38.37	36.81	39.87	33.51	44.26
S3	9.52	8.78	10.59	6.51	13.83	11.72	13.69	9.44	8.52	10.66	5.67	15.5
S4	1.82	1.62	2.07	1	3.85	2.46	3.85	1.87	1.63	2.09	1.01	3.76
AF	3.63	3.04	4.42	1.16	7.54	5.24	7.54	3.77	3.14	4.51	1.11	8.42
**B**	7.01	6.43	8.05	3.95	11.44	5.01	3.95	7.29	6.46	8.35	3.83	12.03
CF	35.43	32.69	37.77	22.72	46.92	26.51	24.8	34.81	31.78	37.54	22.86	44.41

G0	6.33	5.35	7.24	3.01	15.06	5.51	4.09	6.61	5.4	7.87	3.21	12.07
**G1**	8.95	7.84	9.8	4.8	14.4	6.06	5.6	8.13	7	9.24	4.32	12.67
G2	66.29	64.86	67.65	58.35	71.86	67.12	66.13	66.79	65.35	68.22	60.79	71.17
G3	13.67	12.54	15.28	9.45	20.49	16.53	18.13	13.34	12.2	15.25	8.52	21.89
G4	2.31	2.09	2.64	1.33	4.78	3.1	4.78	2.38	2.11	2.64	1.32	4.72

Bolded are derived glycan traits that significantly differed between healthy and diseased siblings. Ctrl$_1$, control sample of individual that developed type 1 diabetes within nearly 6 years; Ctrl$_2$, control sample of individual that developed type 1 diabetes within 9 years; GP, glycan peak; Med, median; AF, glycans with antennary fucose; B, glycans with bisecting *N*-acetylglucosamine (GlcNAc); CF, glycans with core fucose; G0, agalactosylated glycans; G1, monogalactosylated glycans; G2, digalactosylated glycans; G3, trigalactosylated glycans; G4, tetragalactosylated glycans; HB, highly branched glycans; HM, high-mannose glycans; LB, low branched glycans; S0, asialylated glycans; S1, monosialylated glycans; S2, disialylated glycans; S3, trisialylated glycans; S4, tetrasialylated glycans

ESM Table 7 Descriptive statistics of IgG *N*-glycans quantified in the follow-up family-based study

Control						Ctrl$_1$	Ctrl$_2$	Cases				
**GP**	**Med**	**Q1**	**Q3**	**Min**	**Max**	**Area**	**Area**	**Med**	**Q1**	**Q3**	**Min**	**Max**
GP1	0.09	0.07	0.1	0.04	0.21	0.1	0.09	0.09	0.07	0.1	0.04	0.4
GP2	0.24	0.18	0.35	0.07	1.09	0.43	0.23	0.25	0.16	0.39	0.06	0.98
GP3	0.14	0.12	0.17	0.07	0.28	0.13	0.11	0.14	0.12	0.17	0.08	0.29
GP4	19.69	16.64	22.41	9.08	35.05	22.42	20.53	21.06	17.94	24.17	11.45	33.89
GP5	0.17	0.15	0.19	0.1	0.34	0.25	0.11	0.18	0.16	0.21	0.12	0.36
GP6	3.38	2.9	3.86	1.65	7.77	3.79	2.25	3.82	3.2	4.4	1.94	6.97
GP7	0.22	0.16	0.3	0.07	0.91	0.27	0.17	0.21	0.15	0.28	0.06	0.85
GP8	22.15	21.1	23.57	16.39	26	21.67	23.3	21.05	20.03	22.34	17.11	25.38
GP9	8.61	7.91	9.36	5.57	12.18	7.28	9.27	8.29	7.37	9.11	5.43	11.48
GP10	3.7	3.25	4.26	1.89	7.62	3.67	2.15	3.74	3.23	4.38	2.15	6.92
GP11	0.43	0.39	0.49	0.26	0.75	0.43	0.27	0.46	0.42	0.52	0.29	0.79
GP12	0.44	0.31	0.68	0.1	3.33	0.52	0.36	0.4	0.28	0.61	0.14	2.08
GP13	0.25	0.22	0.29	0.15	0.53	0.26	0.2	0.24	0.22	0.28	0.16	0.45
GP14	17.51	15.61	19.32	10.22	25.88	17.73	17.11	17.02	14.93	19.09	9.9	26.4
GP15	1.26	1.07	1.5	0.56	2.35	1.18	0.72	1.31	1.13	1.54	0.78	2.31
GP16	2.67	2.42	2.93	1.65	3.79	2.29	3.19	2.62	2.42	2.83	1.78	3.59
GP17	0.74	0.65	0.84	0.49	1.67	0.76	0.69	0.74	0.66	0.85	0.5	1.57

GP18	11.3	9.87	12.72	6.42	19.3	11.12	13.28	10.96	9.72	12.6	6.66	18.34
GP19	1.42	1.25	1.59	0.75	2.37	1.25	0.96	1.52	1.34	1.69	0.96	3.39
GP20	0.25	0.21	0.29	0.11	0.55	0.24	0.26	0.26	0.22	0.31	0.11	0.66
GP21	0.55	0.51	0.61	0.38	0.83	0.53	0.59	0.57	0.51	0.63	0.4	1.45
GP22	0.17	0.15	0.19	0.09	0.31	0.16	0.17	0.18	0.15	0.21	0.08	0.49
GP23	2.06	1.86	2.4	0.96	4.36	2	2.76	2.17	1.91	2.47	1.08	4.2
GP24	1.35	1.2	1.55	0.7	2.24	1.25	1.03	1.5	1.32	1.75	0.86	2.5
**S0**	55.24	53.18	57.43	41.74	64.38	53.02	53.54	53.2	50.85	55.39	44.77	64.08
S1	16.2	14.69	17.68	10.77	24.36	15.43	18.12	16.03	14.45	17.69	10.77	22.37
**S2**	4.22	3.81	4.6	2.67	7.15	3.93	4.55	4.44	4.03	5.01	2.54	7.43
**B**	12.1	10.93	13.35	6.96	20.84	11.98	7.74	12.89	11.71	14.23	8.61	19.92
CF	96.65	96	97.2	91.15	98.3	96.18	96.89	96.62	96.08	97.15	93.51	98.44
G0	23.46	20.02	26.46	11.83	43.44	26.75	23.09	25.51	22.09	28.79	14.16	39.31
**G1**	38.47	37.05	39.58	31.56	42.39	35.62	38.34	36.49	35.2	38.13	30.27	40.86
G2	37.61	33.94	41.19	23.55	50.91	36.76	37.87	36.91	33.07	41.15	22.96	48.84

Bolded are derived glycan traits that significantly differed between healthy and diseased siblings. $Ctrl_1$, control sample of individual that developed type 1 diabetes within nearly 6 years; $Ctrl_2$, control sample of individual that developed type 1 diabetes within 9 years; GP, glycan peak; Med, median; B, glycans with bisecting *N*-acetylglucosamine (GlcNAc); CF, glycans with core fucose; G0, agalactosylated glycans; G1, monogalactosylated glycans; G2, digalactosylated glycans; S0, asialylated glycans; S1, monosialylated glycans; S2, disialylated glycans

ESM Table 8 Correlation of plasma *N*-glycans with increase in number of specific autoantibodies (1–4)

**Glycan peak**	**Beta**	**SE**	**95% CI_{low}**	**95% CI_{up}**	**T value**	***p* value**	**p_{adj}**	**p_{lab}**
GP1	0.43	0.20	0.04	0.82	2.19	2.90×10^{-2}	1	
GP2	0.54	0.19	0.16	0.92	2.82	5.09×10^{-3}	1	
GP3	-0.02	0.18	-0.38	0.33	-0.12	9.02×10^{-1}	1	
GP4	0.43	0.19	0.05	0.81	2.23	2.63×10^{-2}	1	
GP5	0.48	0.18	0.13	0.84	2.68	7.79×10^{-3}	1	
GP6	0.49	0.18	0.13	0.84	2.68	7.76×10^{-3}	1	
GP7	0.26	0.19	-0.11	0.64	1.37	1.71×10^{-1}	1	

GP8	-0.09	0.20	-0.47	0.30	-0.43	6.66×10^{-1}	1	
GP9	0.04	0.20	-0.35	0.43	0.21	8.35×10^{-1}	1	
GP10	0.37	0.20	-0.02	0.76	1.87	6.31×10^{-2}	1	
GP11	0.34	0.18	-0.01	0.68	1.93	5.51×10^{-2}	1	
GP12	-0.01	0.19	-0.38	0.35	-0.06	9.55×10^{-1}	1	
GP13	0.62	0.19	0.25	0.99	3.28	1.15×10^{-3}	4.35×10^{-1}	
GP14	-0.22	0.20	-0.61	0.16	-1.13	2.59×10^{-1}	1	
GP15	0.26	0.20	-0.13	0.65	1.31	1.91×10^{-1}	1	
GP16	0.54	0.20	0.16	0.93	2.77	6.01×10^{-3}	1	
GP17	0.38	0.18	0.04	0.73	2.18	2.99×10^{-2}	1	
GP18	-0.51	0.17	-0.85	-0.16	-2.89	4.20×10^{-3}	1	
GP19	-0.07	0.20	-0.47	0.32	-0.36	7.17E $\times 10^{-1}$	1	
GP20	-0.28	0.20	-0.67	0.11	-1.42	1.58×10^{-1}	1	
GP21	-0.06	0.20	-0.45	0.34	-0.28	7.81×10^{-1}	1	
GP22	0.40	0.19	0.02	0.77	2.06	4.02×10^{-2}	1	
GP23	0.48	0.18	0.13	0.83	2.67	7.99×10^{-3}	1	
GP24	-0.87	0.19	-1.24	-0.50	-4.65	5.23×10^{-6}	1.98×10^{-3}	**
GP25	-0.50	0.20	-0.89	-0.12	-2.56	1.10×10^{-2}	1	
GP26	-0.71	0.20	-1.09	-0.32	-3.60	3.72×10^{-4}	1	
GP27	0.27	0.20	-0.12	0.65	1.35	1.77×10^{-1}	1	
GP28	-0.86	0.18	-1.22	-0.50	-4.66	4.85×10^{-6}	1.83×10^{-3}	**
GP29	-0.30	0.19	-0.69	0.08	-1.57	1.18×10^{-1}	1	
GP30	-0.93	0.19	-1.30	-0.56	-4.99	1.07×10^{-6}	4.04×10^{-4}	***
GP31	-0.52	0.20	-0.91	-0.13	-2.63	8.93×10^{-3}	1	
GP32	-0.32	0.20	-0.71	0.06	-1.64	1.02×10^{-1}	1	
GP33	0.13	0.19	-0.26	0.51	0.65	5.13×10^{-1}	1	
GP34	-0.64	0.19	-1.02	-0.26	-3.33	9.99×10^{-4}	3.78×10^{-1}	
GP35	0.22	0.20	-0.17	0.61	1.13	2.59×10^{-1}	1	
GP36	-0.46	0.19	-0.84	-0.07	-2.35	1.97×10^{-2}	1	
GP37	-0.78	0.18	-1.13	-0.42	-4.34	1.98×10^{-5}	7.50×10^{-3}	**
GP38	-0.66	0.18	-1.02	-0.30	-3.58	4.05×10^{-4}	1.53×10^{-1}	
GP39	-0.08	0.19	-0.46	0.30	-0.42	6.73×10^{-1}	1	
LB	0.69	0.19	0.32	1.06	3.66	3.07×10^{-4}	1.16×10^{-1}	
HB	-0.77	0.19	-1.14	-0.40	-4.13	4.72×10^{-5}	1.79×10^{-2}	*
G0	0.46	0.20	0.08	0.85	2.36	1.90×10^{-2}	1	
G1	0.53	0.19	0.16	0.90	2.80	5.49×10^{-3}	1	
G2	0.11	0.20	-0.28	0.50	0.55	5.81×10^{-1}	1	
G3	-0.79	0.19	-1.15	-0.42	-4.19	3.76×10^{-5}	1.42×10^{-2}	*
G4	-0.60	0.18	-0.96	-0.24	-3.25	1.30×10^{-3}	4.93×10^{-1}	
S1	0.35	0.20	-0.04	0.74	1.75	8.08×10^{-2}	1	
S2	-0.46	0.19	-0.85	-0.08	-2.39	1.75×10^{-2}	1	
S3	-0.75	0.19	-1.12	-0.38	-4.00	8.13×10^{-5}	3.07×10^{-2}	*
S4	-0.60	0.18	-0.96	-0.24	-3.28	1.15×10^{-3}	4.36×10^{-1}	
B	0.58	0.17	0.25	0.92	3.41	7.48×10^{-4}	2.83×10^{-1}	
AF	0.15	0.20	-0.24	0.53	0.75	4.54×10^{-1}	1	
CF	0.61	0.19	0.25	0.98	3.29	1.13×10^{-3}	4.27×10^{-1}	

HM	0.10	0.20	-0.29	0.49	0.50	6.16×10^{-1}	1	

Beta, standard error, and 95% confidence interval are expressed in the standard deviation units, adjusted for age and gender. Significant associations are represented with a star sign within p_{lab} column.

LB, low branched glycans; HB, highly branched glycans; G0, agalactosylated glycans; G1, monogalactosylated glycans; G2, digalactosylated glycans; G3, trigalactosylated glycans; G4, tetragalactosylated glycans; S1, monosialylated glycans; S2, disialylated glycans; S3, trisialylated glycans; S4, tetrasialylated glycans; B, glycans with bisecting *N*-acetylglucosamine (GlcNAc); AF, glycans with antennary fucose; CF, glycans with core fucose; HM, high-mannose glycans; p_{adj}, *p* value adjusted using Benjamini-Hochberg's method; p_{lab}, labels for significant *p* values, p $value_{adj}$ < 0.05 (*), p $value_{adj}$ < 0.01 (**), p $value_{adj}$ < 0.001 (***)

ESM Table 9 Associations of plasma *N*-glycans with disease status, further adjusted for age and sex, and corrected for multiple testing

**Glycan**	**OR**	**95% CI**	**SE**	***p* value**
GP1	1.07	0.83, 1.39	0.14	7.93×10^{-1}
GP2	2.09	1.58, 2.76	0.30	**1.16×10^{-5}**
GP3	0.87	0.64, 1.19	0.14	6.27×10^{-1}
GP4	0.60	0.46, 0.79	0.08	**2.16×10^{-3}**
GP5	0.64	0.47, 0.86	0.10	**1.32×10^{-2}**
GP6	0.97	0.73, 1.3	0.14	9.60×10^{-1}
GP7	1.69	1.28, 2.24	0.24	**2.01×10^{-3}**
GP8	1.03	0.8, 1.33	0.13	9.60×10^{-1}
GP9	0.95	0.7, 1.27	0.14	9.19×10^{-1}
GP10	0.57	0.43, 0.76	0.08	**1.24×10^{-3}**
GP11	1.27	0.93, 1.75	0.21	2.92×10^{-1}
GP12	1.86	1.37, 2.53	0.29	**1.15×10^{-3}**
GP13	0.87	0.66, 1.14	0.12	5.16×10^{-1}
GP14	1.18	0.91, 1.54	0.16	3.78×10^{-1}
GP15	1.13	0.85, 1.49	0.16	6.27×10^{-1}
GP16	1.01	0.78, 1.31	0.13	9.89×10^{-1}
GP17	1.47	1.08, 2	0.23	**5.04×10^{-2}**
GP18	1.04	0.79, 1.39	0.15	9.59×10^{-1}
GP19	1.00	0.78, 1.29	0.13	9.89×10^{-1}
GP20	0.98	0.76, 1.28	0.13	9.89×10^{-1}
GP21	1.52	1.16, 1.98	0.21	**8.56×10^{-3}**
GP22	1.74	1.32, 2.29	0.25	**1.15×10^{-3}**
GP23	1.66	1.23, 2.25	0.26	**5.61×10^{-3}**
GP24	0.79	0.61, 1.02	0.10	1.89×10^{-1}
GP25	2.12	1.56, 2.87	0.33	**4.54×10^{-5}**
GP26	0.82	0.63, 1.06	0.11	2.92×10^{-1}
GP27	0.99	0.77, 1.29	0.13	9.89×10^{-1}
GP28	0.97	0.74, 1.26	0.13	9.60×10^{-1}
GP29	1.62	1.2, 2.19	0.25	**7.12×10^{-3}**
GP30	0.72	0.55, 0.94	0.10	5.14×10^{-2}

GP31	1.20	0.92, 1.56	0.16	3.40 x 10^{-1}
GP32	0.74	0.57, 0.96	0.10	7.91 x 10^{-2}
GP33	1.00	0.77, 1.29	0.13	9.89 x 10^{-1}
GP34	1.10	0.84, 1.43	0.15	7.23 x 10^{-1}
GP35	1.30	0.99, 1.7	0.18	1.62 x 10^{-1}
GP36	1.08	0.82, 1.41	0.15	7.93 x 10^{-1}
GP37	0.86	0.65, 1.14	0.12	5.06 x 10^{-1}
GP38	0.91	0.68, 1.2	0.13	7.23 x 10^{-1}
GP39	0.97	0.74, 1.27	0.13	9.60 x 10^{-1}
AF	1.01	0.78, 1.31	0.13	9.89 x 10^{-1}
B	1.72	1.26, 2.34	0.27	**3.52 x 10^{-3}**
CF	1.01	0.77, 1.32	0.14	9.89 x 10^{-1}
G0	1.23	0.95, 1.6	0.16	2.73 x 10^{-1}
G1	0.63	0.47, 0.84	0.09	**7.01 x 10^{-3}**
G2	1.24	0.96, 1.6	0.16	2.61 x 10^{-1}
G3	0.81	0.62, 1.05	0.11	2.73 x 10^{-1}
G4	0.93	0.7, 1.23	0.13	7.93 x 10^{-1}
HB	0.83	0.64, 1.08	0.11	3.32 x 10^{-1}
HM	1.65	1.26, 2.16	0.23	**2.16 x 10^{-3}**
LB	1.15	0.88, 1.5	0.15	5.07 x 10^{-1}
S1	1.33	1.02, 1.72	0.18	9.92 x 10^{-2}
S2	1.18	0.91, 1.54	0.16	3.78 x 10^{-1}
S3	0.83	0.64, 1.08	0.11	3.32 x 10^{-1}
S4	0.89	0.67, 1.19	0.13	6.60 x 10^{-1}

Significant *p* values ($p < 0.05$) are represented in bold.

LB, low branched glycans; HB, highly branched glycans; G0, agalactosylated glycans; G1, monogalactosylated glycans; G2, digalactosylated glycans; G3, trigalactosylated glycans; G4, tetragalactosylated glycans; S1, monosialylated glycans; S2, disialylated glycans; S3, trisialylated glycans; S4, tetrasialylated glycans; B, glycans with bisecting N-acetylglucosamine (GlcNAc); AF, glycans with antennary fucose; CF, glycans with core fucose; HM, high-mannose glycans

ESM Table 10 Associations of IgG *N*-glycans with disease status, further adjusted for age and sex, and corrected for multiple testing

**Glycan**	**OR**	**95% CI**	**SE**	***p* value**
GP1	0.79	0.6, 1.04	0.11	1.57 x 10^{-1}
GP2	1.15	0.88, 1.51	0.16	3.43 x 10^{-1}
GP3	0.86	0.66, 1.12	0.12	3.43 x 10^{-1}
GP4	1.25	0.95, 1.63	0.17	1.57 x 10^{-1}
GP5	1.53	1.17, 1.99	0.21	**3.89 x 10^{-3}**
GP6	1.71	1.31, 2.24	0.23	**3.02 x 10^{-4}**
GP7	1.00	0.76, 1.31	0.14	9.73 x 10^{-1}
GP8	0.40	0.3, 0.54	0.06	**7.95 x 10^{-9}**
GP9	0.69	0.52, 0.93	0.10	**2.73 x 10^{-2}**

GP10	1.32	1, 1.74	0.19	8.64×10^{-2}
GP11	1.90	1.43, 2.54	0.28	**4.26×10^{-5}**
GP12	0.98	0.74, 1.3	0.14	9.64×10^{-1}
GP13	1.01	0.77, 1.31	0.14	9.73×10^{-1}
GP14	0.85	0.66, 1.1	0.11	2.97×10^{-1}
GP15	2.09	1.52, 2.87	0.34	**3.49×10^{-5}**
GP16	0.81	0.63, 1.05	0.11	1.57×10^{-1}
GP17	1.41	1.07, 1.85	0.20	**2.73×10^{-2}**
GP18	0.87	0.67, 1.13	0.11	3.43×10^{-1}
GP19	2.91	2.09, 4.05	0.49	**4.90×10^{-9}**
GP20	1.60	1.21, 2.1	0.22	**2.29×10^{-3}**
GP21	1.69	1.28, 2.24	0.24	**6.24×10^{-4}**
GP22	1.39	1.07, 1.81	0.19	**2.73×10^{-2}**
GP23	1.24	0.95, 1.62	0.17	1.57×10^{-1}
GP24	2.62	1.94, 3.54	0.40	**4.90×10^{-9}**
B	2.16	1.61, 2.89	0.32	**1.65×10^{-6}**
CF	0.80	0.6, 1.05	0.11	1.57×10^{-1}
G0	1.34	1.02, 1.76	0.19	5.98×10^{-2}
G1	0.37	0.27, 0.51	0.06	**7.95×10^{-9}**
G2	1.02	0.78, 1.32	0.13	9.64×10^{-1}
HM	1.53	1.17, 1.99	0.21	**3.89×10^{-3}**
S0	0.50	0.37, 0.68	0.08	**3.56×10^{-5}**
S1	0.98	0.76, 1.27	0.13	9.64×10^{-1}
S2	1.86	1.41, 2.45	0.26	**4.19×10^{-5}**

Significant *p* values ($p < 0.05$) are represented in bold.

B, glycans with bisecting N-acetylglucosamine (GlcNAc); CF, glycans with core fucose; G0, agalactosylated glycans; G1, monogalactosylated glycans; G2, digalactosylated glycans; S0, asialylated glycans; S1, monosialylated glycans; S2, disialylated glycans

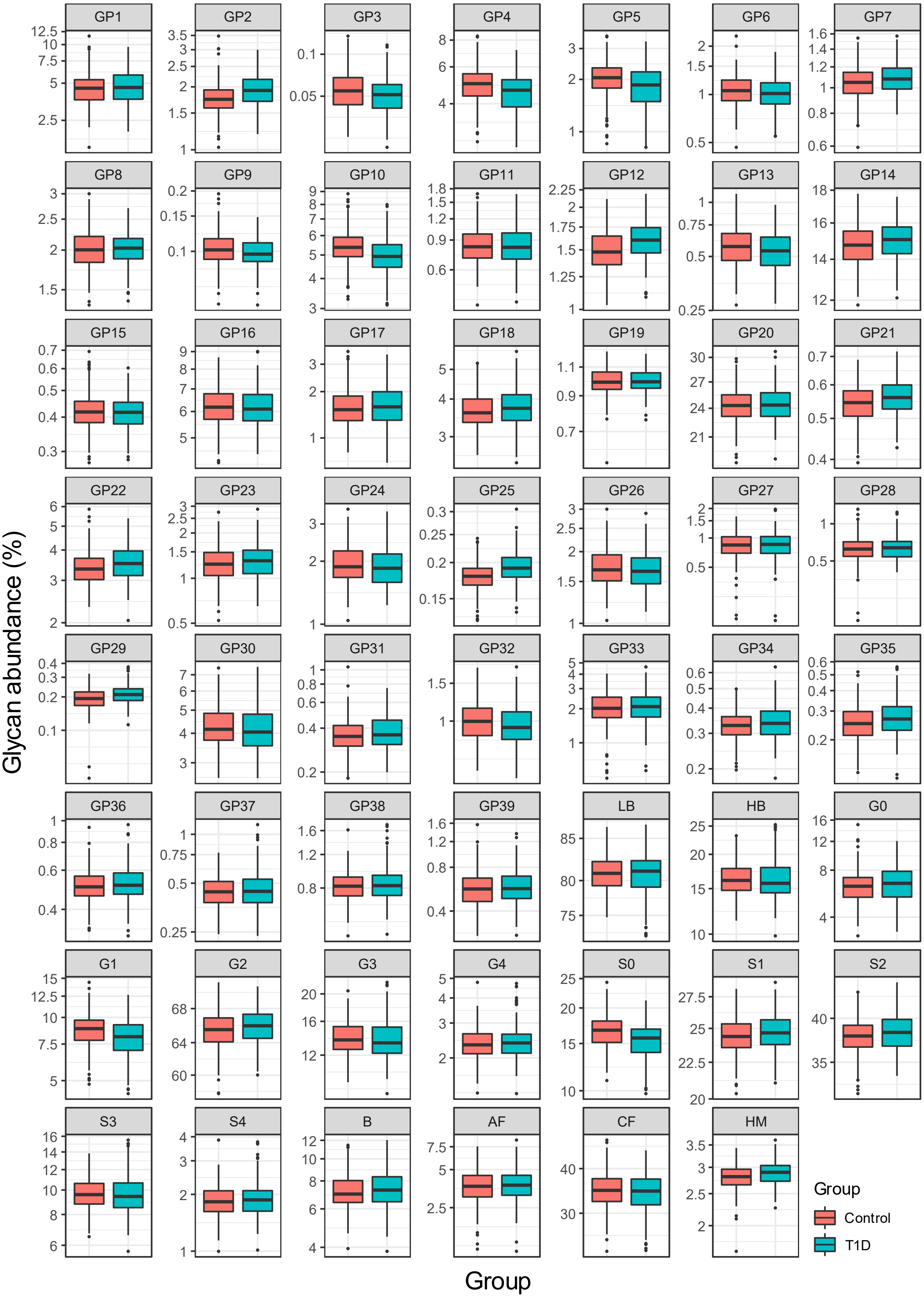

ESM Fig. 1 Plasma *N*-glycan distributions across participants with type 1 diabetes and their unaffected siblings in the follow-up family-based study. GP, glycan peak; AF, glycans with antennary fucose; B, glycans with bisecting *N*-acetylglucosamine (GlcNAc); CF, glycans with core fucose; G0,

agalactosylated glycans; G1, monogalactosylated glycans; G2, digalactosylated glycans; G3, trigalactosylated glycans; G4, tetragalactosylated glycans; HB, highly branched glycans; HM, high-mannose glycans; LB, low branched glycans; S1, monosialylated glycans; S2, disialylated glycans; S3, trisialylated glycans; S4, tetrasialylated glycans

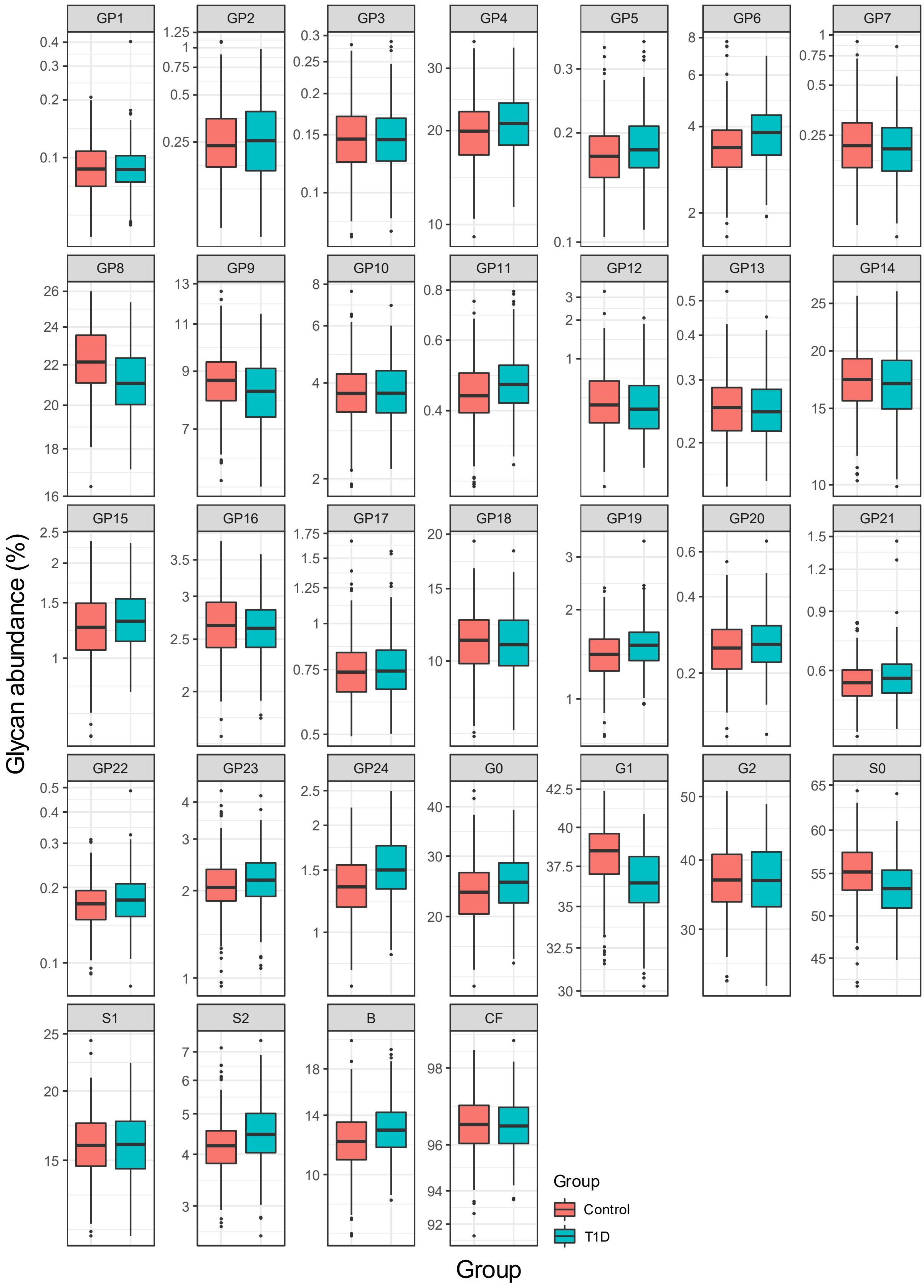

ESM Fig. 2 IgG *N*-glycan distributions across participants with type 1 diabetes and their unaffected siblings in the follow-up family-based study. GP, glycan peak; G0, agalactosylated glycans; G1, monogalactosylated glycans; G2, digalactosylated glycans; S0, asialylated glycans; S1, monosialylated

glycans; S2, disialylated glycans; B, glycans with bisecting N-acetylglucosamine (GlcNAc); CF, glycans with core fucose; HM, high-mannose glycans

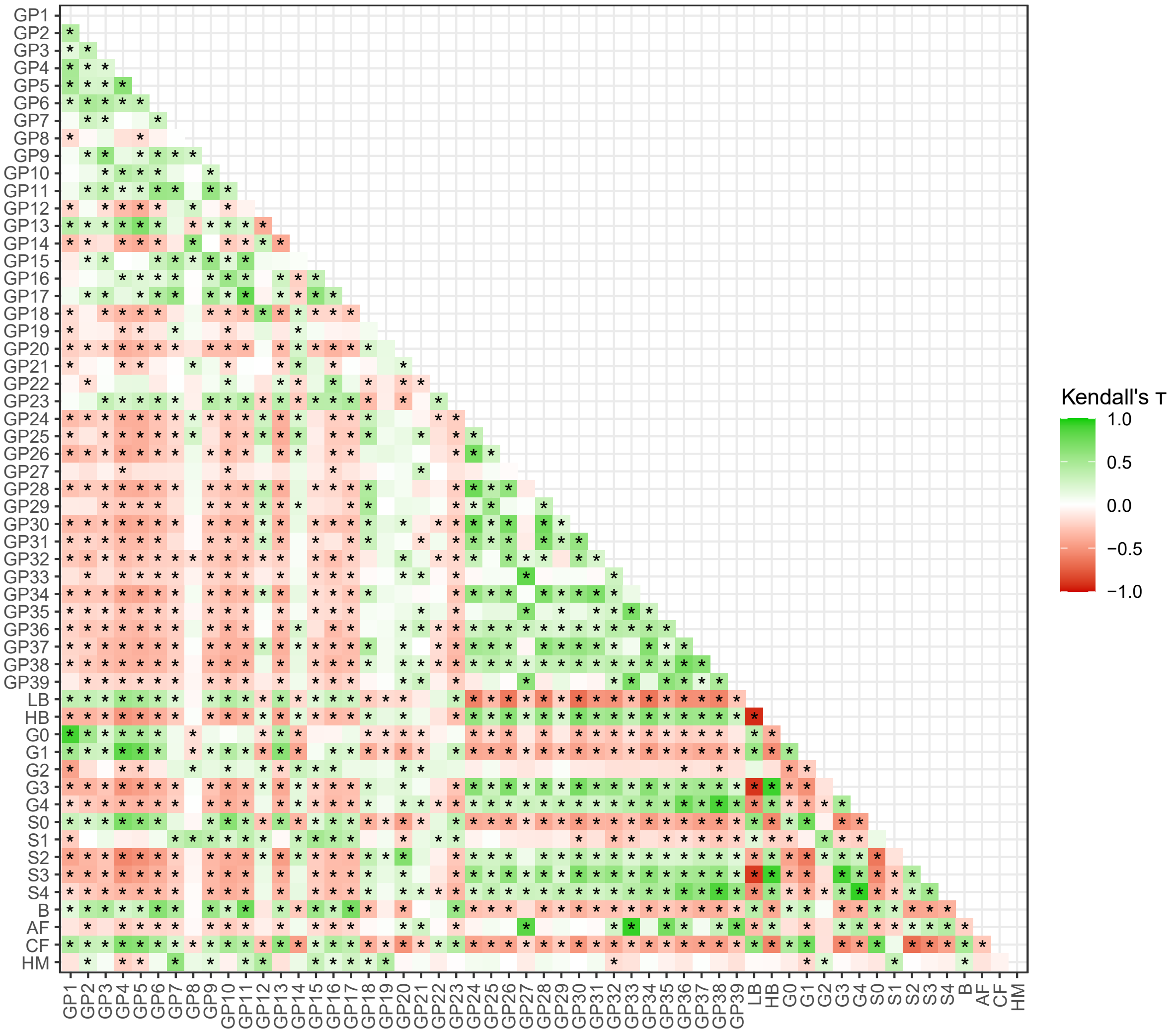

ESM Fig. 3 Intercorrelation of the assessed plasma *N*-glycans across participants with type 1 diabetes from the follow-up family-based study. GP, glycan peak; LB*,* low branched glycans; HB*,* highly branched glycans; G0*,* agalactosylated glycans; G1*,* monogalactosylated glycans; G2*,* digalactosylated glycans; G3*,* trigalactosylated glycans; G4*,* tetragalactosylated glycans; S1*,* monosialylated glycans; S2*,* disialylated glycans; S3*,* trisialylated glycans; S4*,* tetrasialylated glycans; B*,* glycans with bisecting *N*-acetylglucosamine (GlcNAc); AF*,* glycans with antennary fucose; CF*,* glycans with core fucose; HM*,* high-mannose glycans

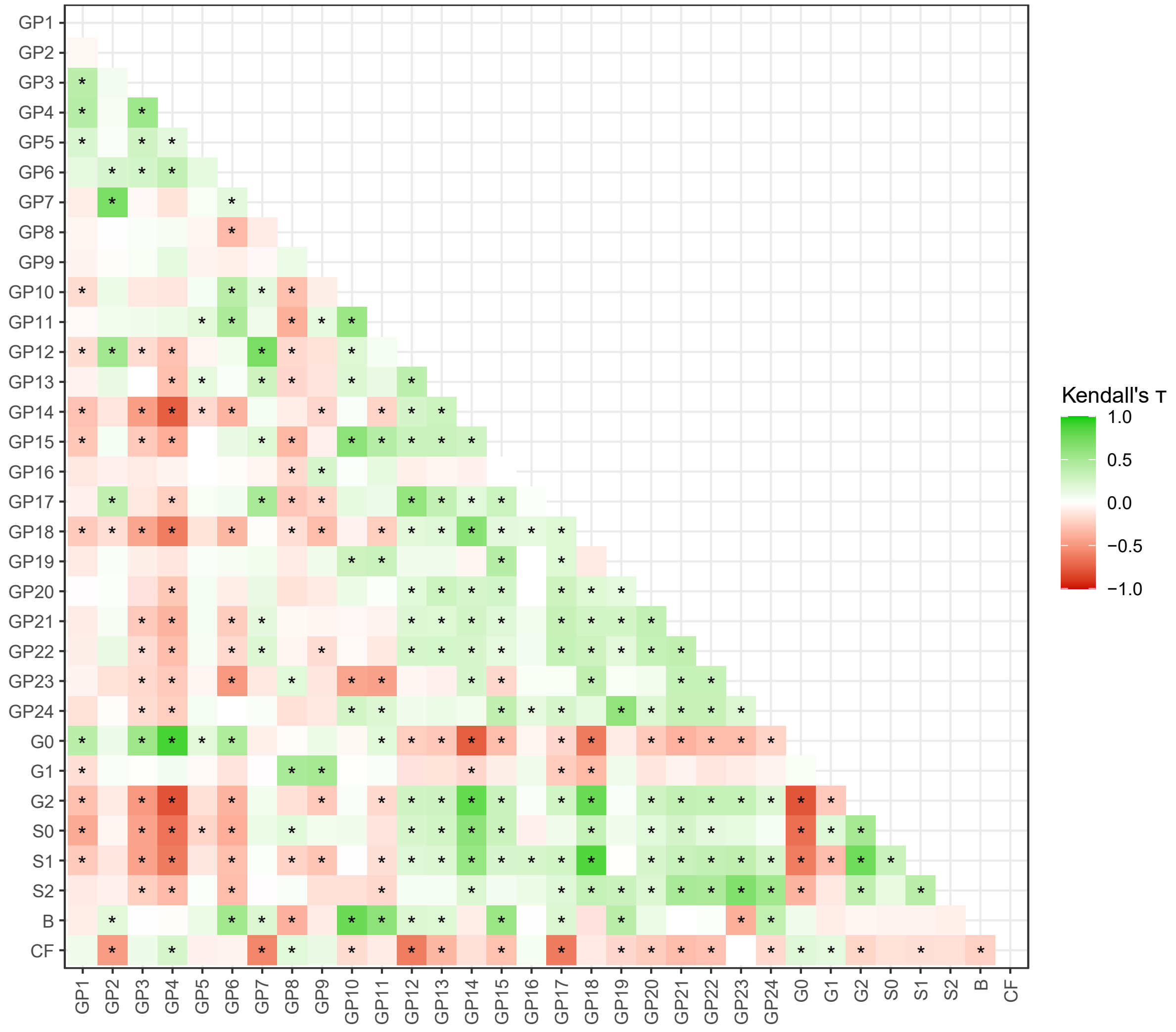

ESM Fig. 4 Intercorrelation of the assessed IgG *N*-glycans across participants with type 1 diabetes from the follow-up family-based study. GP, glycan peak; G0, agalactosylated glycans; G1, monogalactosylated glycans; G2, digalactosylated glycans; S0, asialylated glycans; S1, monosialylated glycans; S2, disialylated glycans; B, glycans with bisecting N-acetylglucosamine (GlcNAc); CF, glycans with core fucose; HM, high-mannose glycans

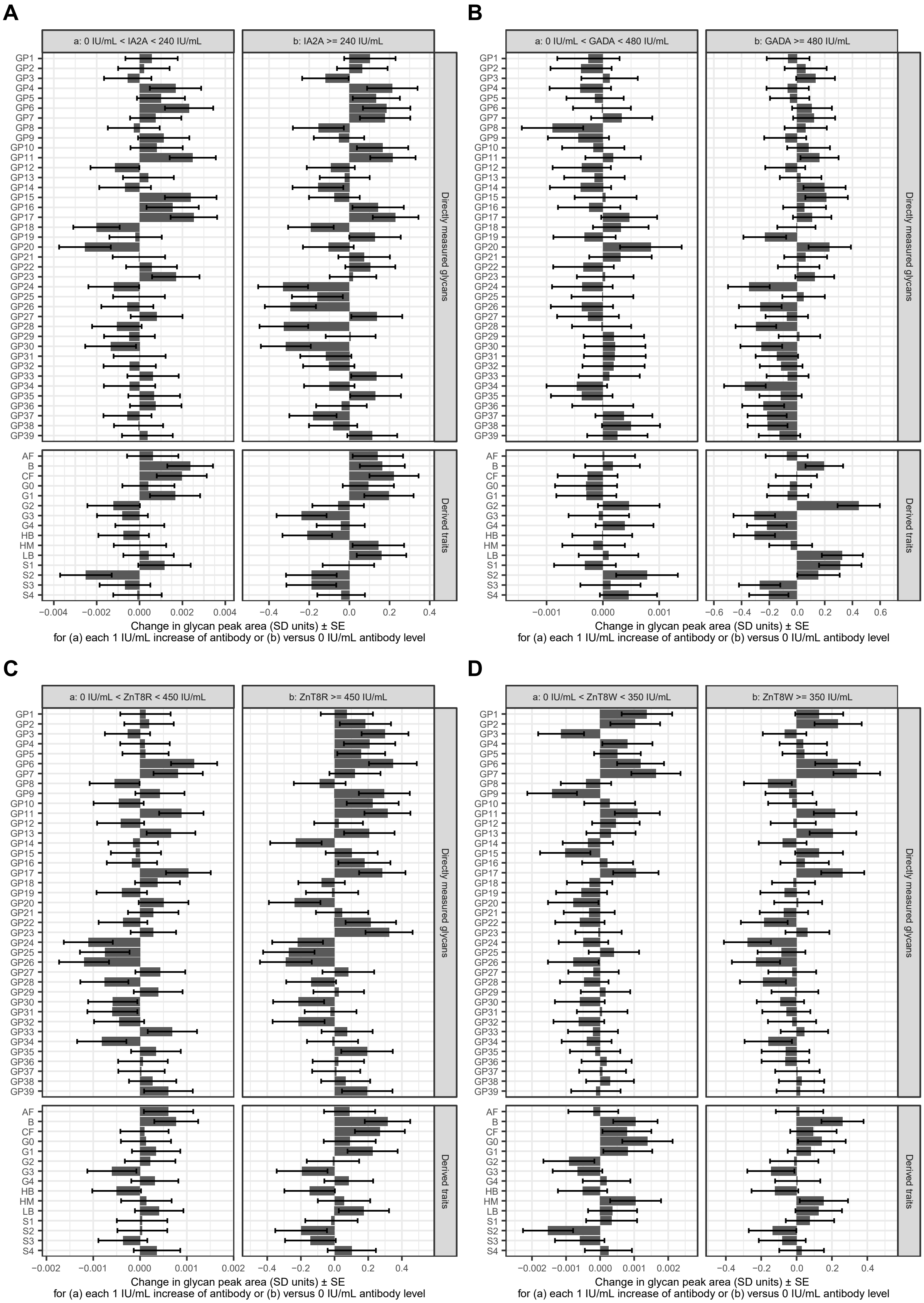

ESM Fig. 5 Associations between plasma *N*-glycans and (A) IA-2A, insulinoma-associated protein 2 autoantibodies; (B) GADA, glutamic acid decarboxylase autoantibodies; (C) ZnT8RA, arginine zinc transporter 8 autoantibodies; (D) ZnT8WA, tryptophan zinc transporter 8 autoantibodies. No

significant associations were observed. LB, low branched glycans; HB, highly branched glycans; G0, agalactosylated glycans; G1, monogalactosylated glycans; G2, digalactosylated glycans; G3, trigalactosylated glycans; G4, tetragalactosylated glycans; S1, monosialylated glycans; S2, disialylated glycans; S3, trisialylated glycans; S4, tetrasialylated glycans; B, glycans with bisecting *N*-acetylglucosamine (GlcNAc); AF, glycans with antennary fucose; CF, glycans with core fucose; HM, high-mannose glycans

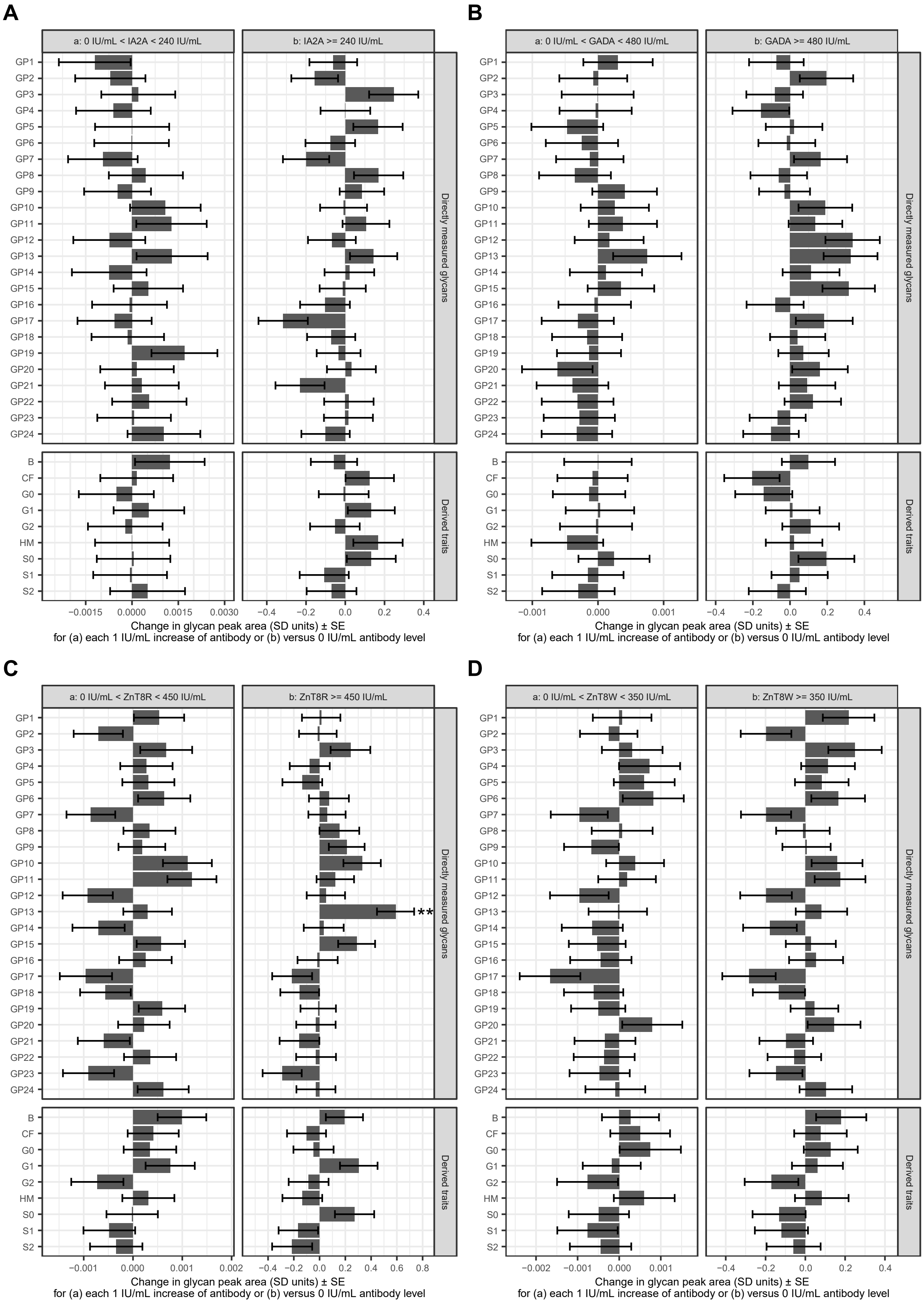

ESM Fig. 6 Associations between IgG *N*-glycans and (A) IA-2A, insulinoma-associated protein 2 autoantibodies; (B) GADA, glutamic acid decarboxylase autoantibodies; (C) ZnT8RA, arginine zinc transporter 8 autoantibodies; (D) ZnT8WA, tryptophan zinc transporter 8 autoantibodies. Significant

associations are represented with a star sign. G0, agalactosylated glycans; G1, monogalactosylated glycans; G2, digalactosylated glycans; S0, asialylated glycans; S1, monosialylated glycans; S2, disialylated glycans; B, glycans with bisecting N-acetylglucosamine (GlcNAc); CF, glycans with core fucose; HM, high-mannose glycans

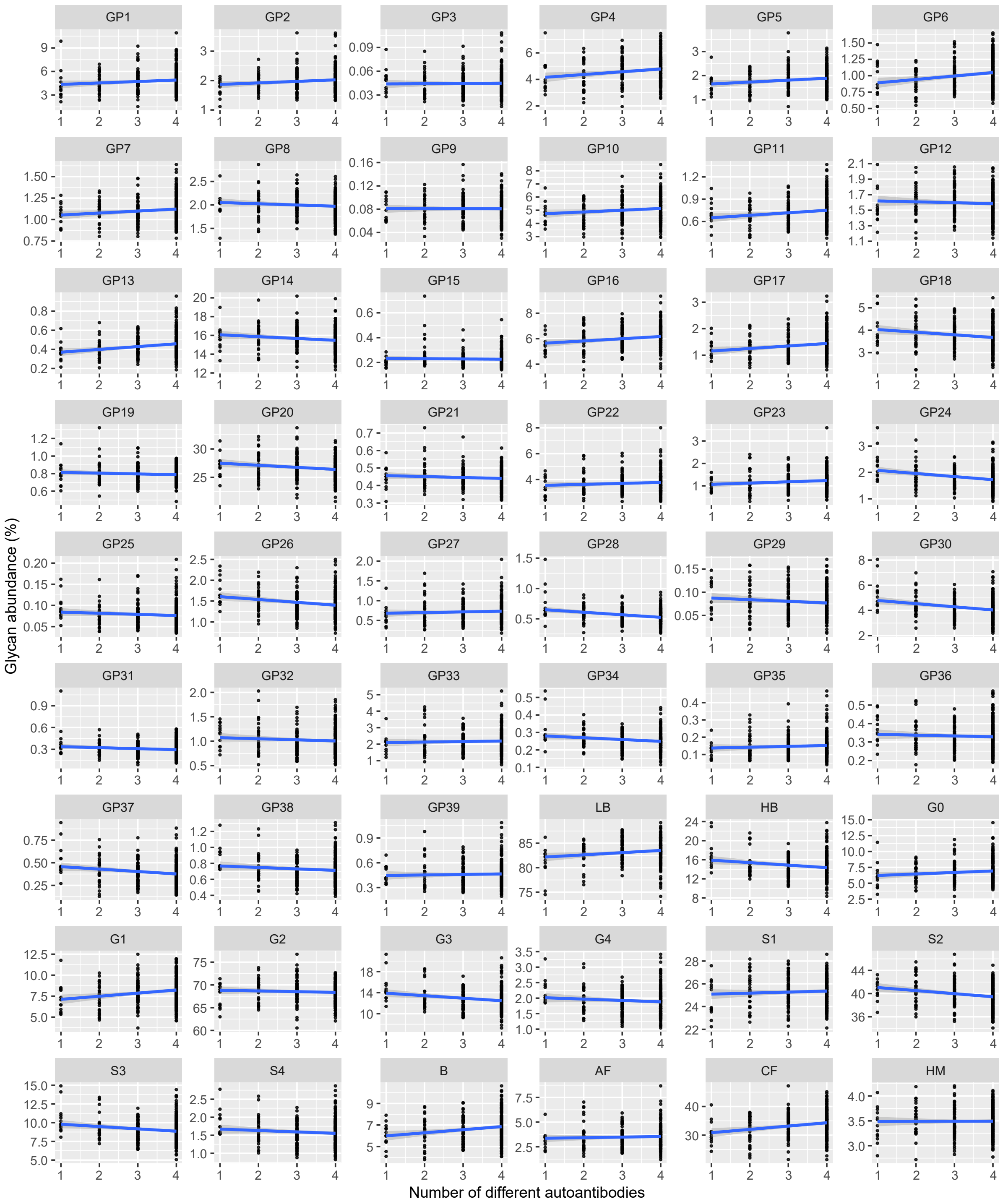

ESM Fig. 7 Correlation of plasma *N*-glycans with increase in number of specific autoantibodies. Significant correlations were observed for GP30, GP24, GP28, GP37, G3, HB, and S3. LB, low branched glycans; HB, highly branched glycans; G0, agalactosylated glycans; G1, monogalactosylated

glycans; G2, digalactosylated glycans; G3, trigalactosylated glycans; G4, tetragalactosylated glycans; S1, monosialylated glycans; S2, disialylated glycans; S3, trisialylated glycans; S4, tetrasialylated glycans; B, glycans with bisecting N-acetylglucosamine (GlcNAc); AF, glycans with antennary fucose; CF, glycans with core fucose; HM, high-mannose glycans

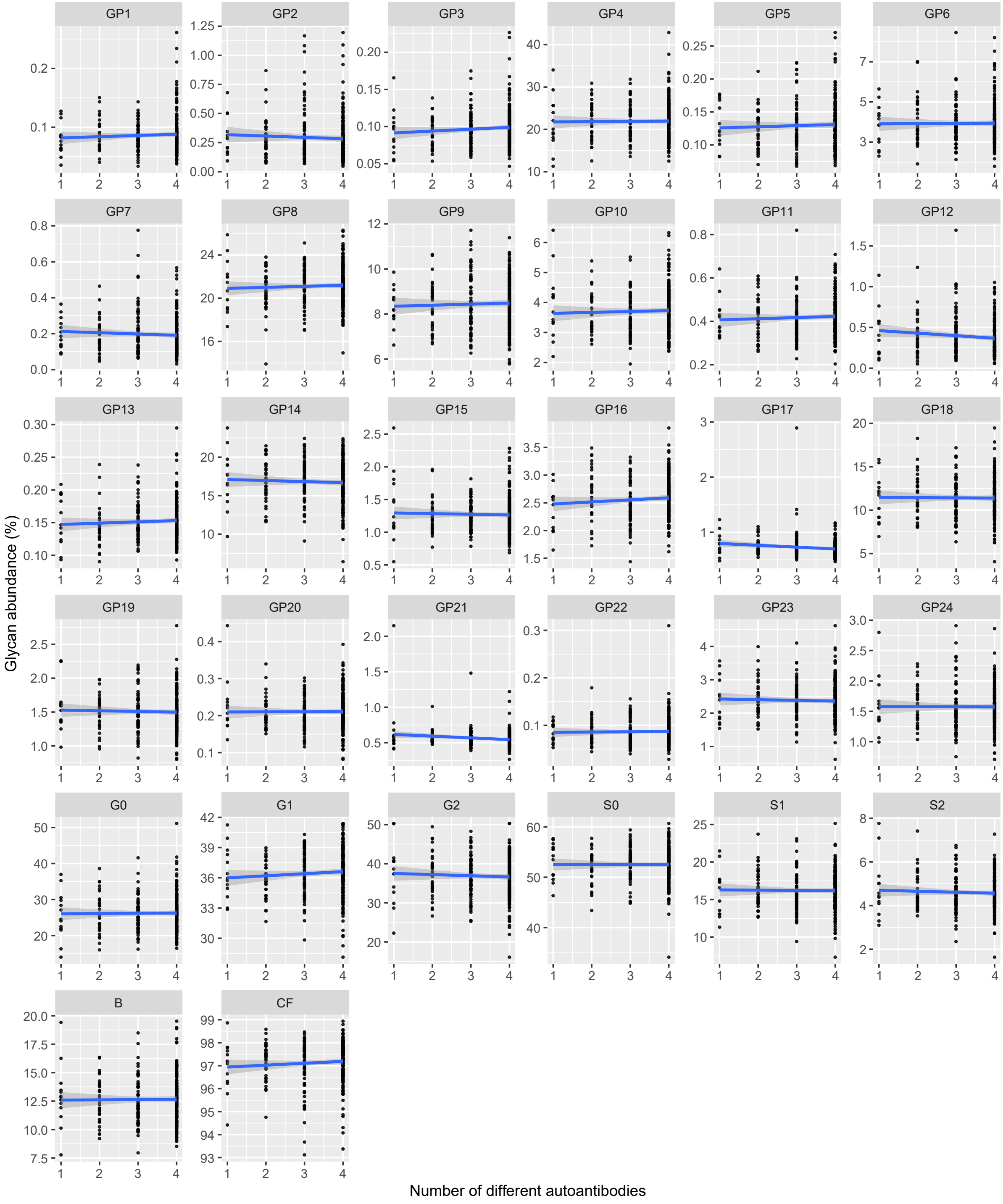

ESM Fig. 8 Correlation of IgG *N*-glycans with increase in number of specific autoantibodies. No significant correlations were observed. G0, agalactosylated glycans; G1, monogalactosylated glycans; G2, digalactosylated glycans; S0, asialylated glycans; S1, monosialylated glycans; S2, disialylated glycans; B, glycans with bisecting N-acetylglucosamine (GlcNAc); CF, glycans with core fucose; HM, high-mannose glycans

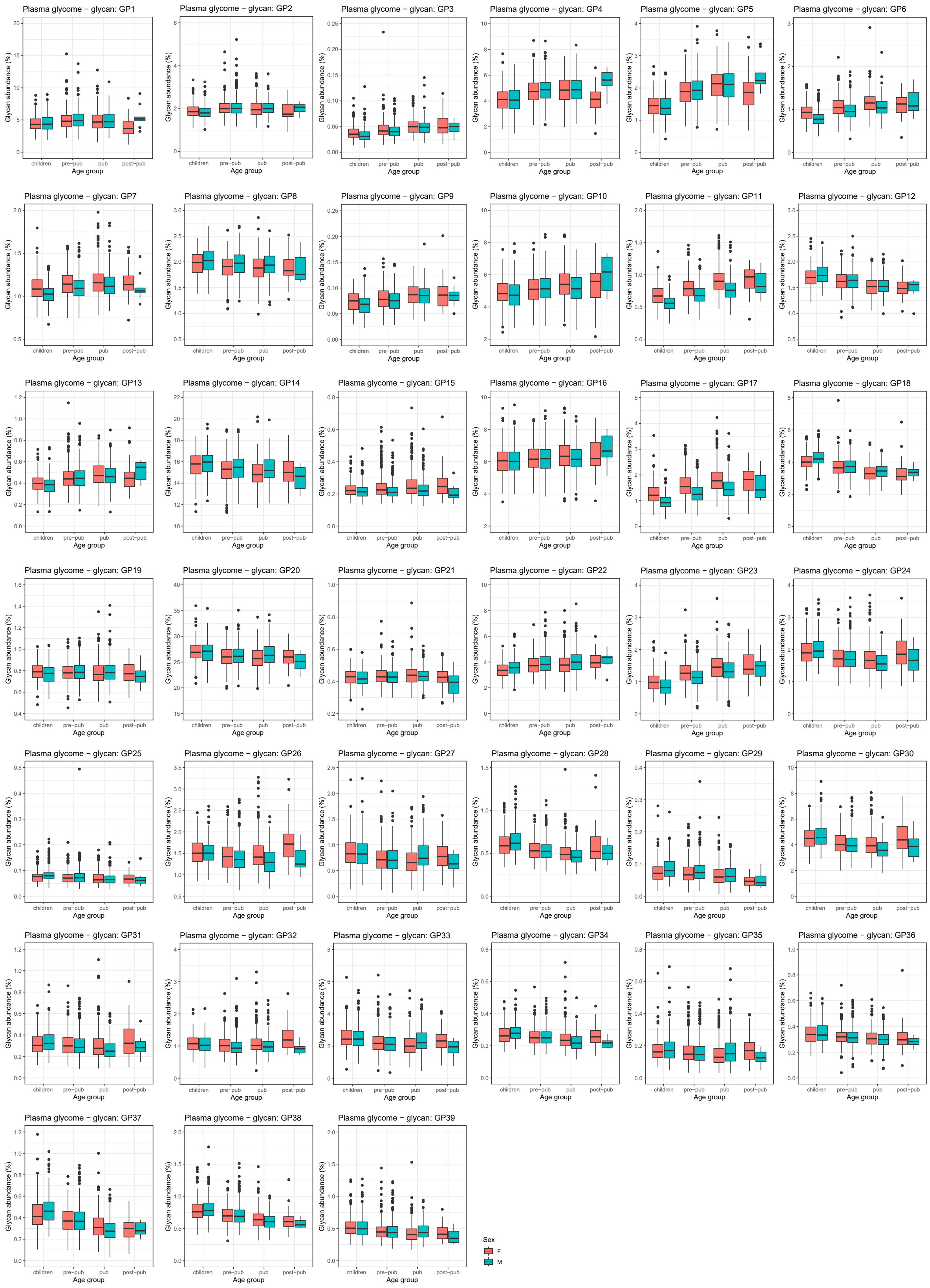

ESM Fig. 9 Proportions of directly measured plasma *N*-glycans in groups comprising male and female children with various ages at diagnosis of type 1 diabetes. Children, age 0.6-6 years; Pre-pub, pre-pubertal children, age 6-11/13 years; Pub, pubertal children, age 11/13-15/16.9 years; Post-pub, post-pubertal children, age 15/17-19.1/18.3 years; F, female; M, male

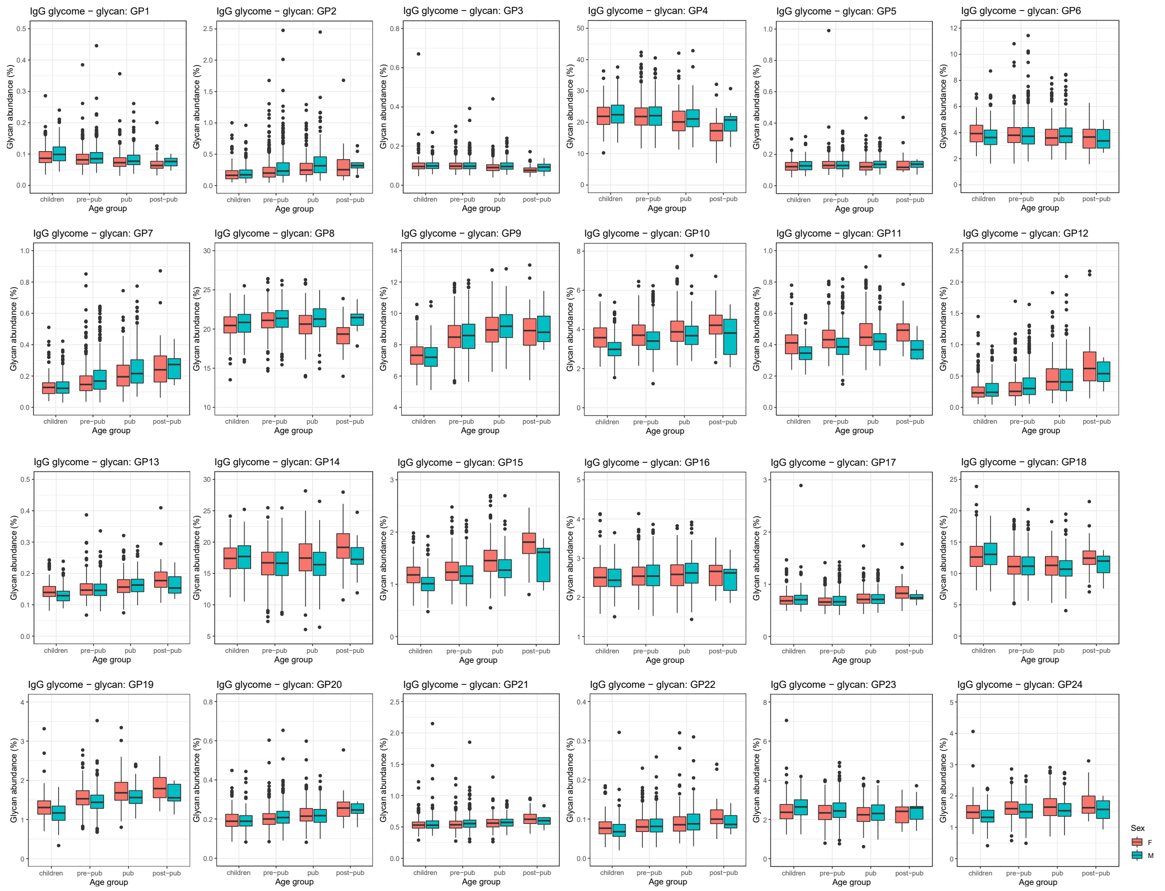

ESM Fig. 10 Proportions of directly measured IgG *N*-glycans in groups comprising male and female children with various ages at diagnosis of type 1 diabetes. Children, age 0.6-6 years; Pre-pub, pre-pubertal children, age 6 to 11/13 years; Pub, pubertal children, age 11/13-15/16.9 years; Post-pub, post-pubertal children, age 15/17-19.1/18.3 years; F, female; M, male

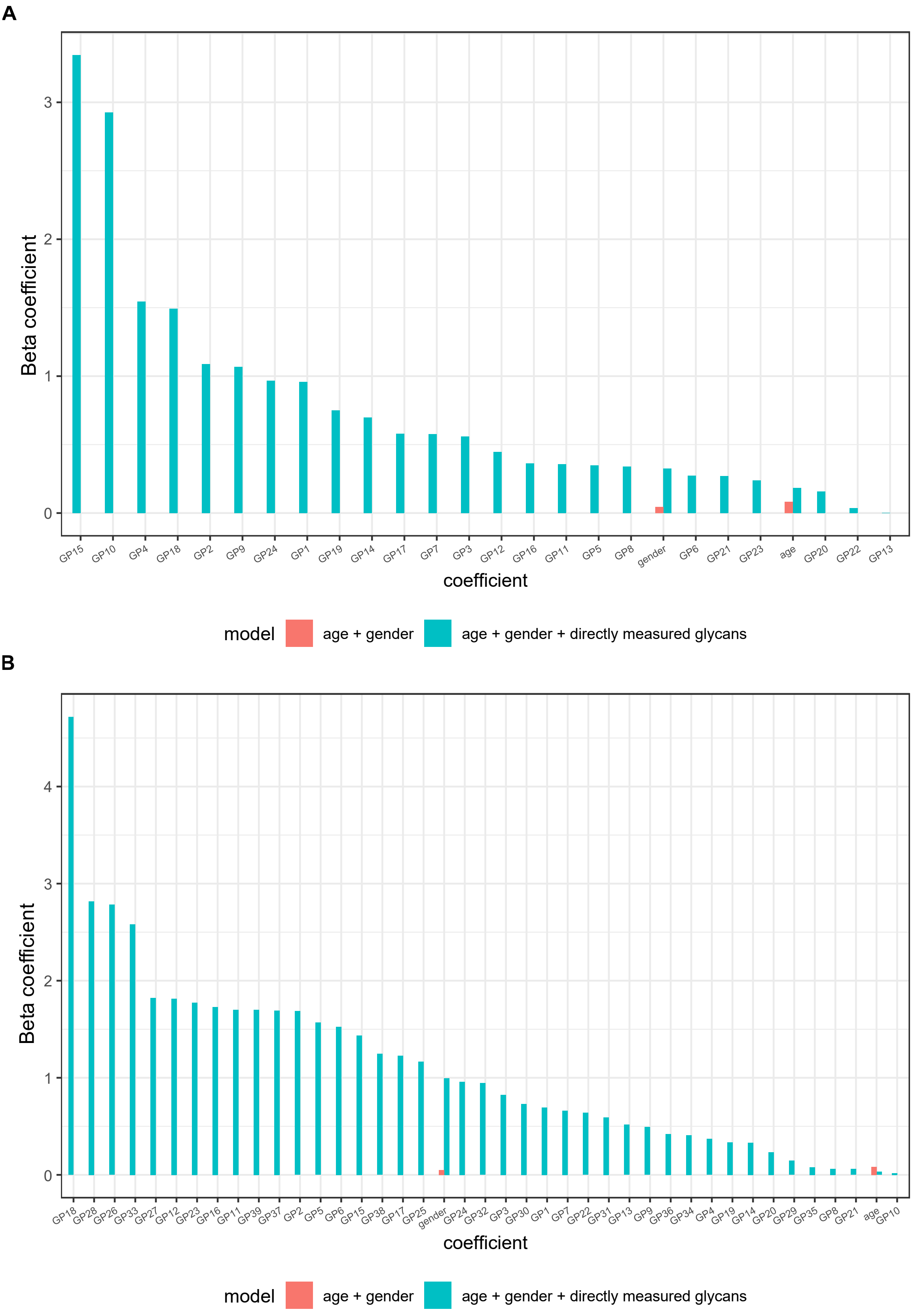

ESM Fig. 11 Classification performance of each individual IgG (A) and plasma (B) *N*-glycan trait in predicting disease status of patients with type 1 diabetes and healthy controls evaluated by the receiver operating characteristic curves analyses
